# Computational Design of an Electro-Organocatalyst for Conversion of CO_2_ into Long Chain Aldehydes

**DOI:** 10.1021/acs.jpca.4c00780

**Published:** 2024-07-04

**Authors:** Foroogh Khezeli, Craig Plaisance

**Affiliations:** † Cain Department of Chemical Engineering, Louisiana State University, Baton Rouge, Louisiana 70803, United States

## Abstract

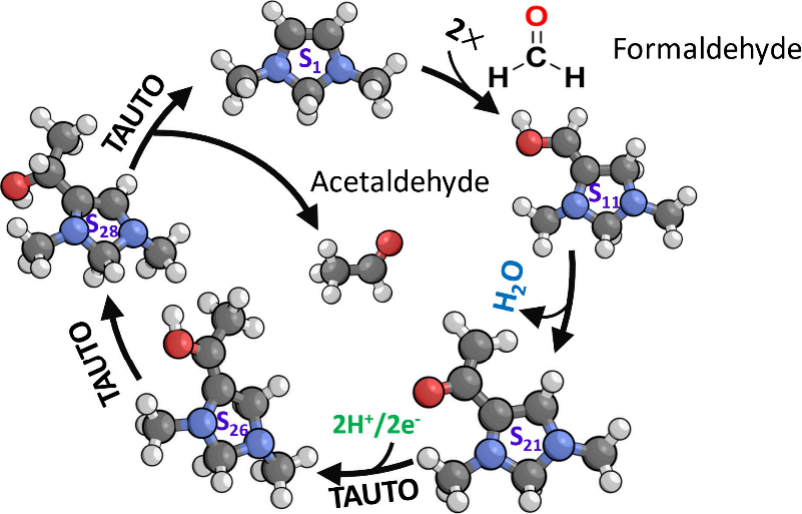

Density functional theory calculations employing a hybrid implicit/explicit solvation method were used to demonstrate that an electro-organocatalyst designed in our previous work for reducing CO_2_ to formaldehyde could also be capable of coupling formaldehyde to form long chain aldehydes. The catalytic activity is enabled by an electron-rich vicinal enediamine (>N–C=C–N<) backbone that activates formaldehyde by reversing the polarity on the carbon atom, enabling it to act as a nucleophile in the subsequent aldol addition step. The catalyst then enables reductive dehydroxylation of the aldol addition product by facilitating outer-sphere electron transfer. The optimal pH as well as the limiting potential and formaldehyde concentration are identified and related to the kinetic balance between several rate limiting steps. Finally, the optimal conditions for coupling with the CO_2_ reduction cycle are discussed, demonstrating that the electro-organocatalyst is capable of efficiently converting CO_2_ into aldehyde products with a turnover frequency (per carbon atom) on the order of 0.1–1 s^–1^.

## 1 Introduction

One of the most significant obstacles toward a societal transition to renewable energy is the lack of an efficient and cost-effective means of storing this energy during times of surplus production and releasing it during times of surplus demand.^1,2^ Electrocatalytic reduction of CO_2_ to liquid transportation fuels or chemical feedstocks could serve this purpose if the process could be improved in terms of cost and efficiency.^3^ The largest impediment toward wider commercialization of this technology is the lack of a suitable electrocatalyst capable of efficiently reducing CO_2_ at the cathode.^4−6^ Existing electrocatalysts exhibit limitations such as low Faradaic efficiency, low current densities, high overpotentials, or insufficient selectivity toward carbon products other than CO, hindering their practical implementation.^7^

**Scheme 1:**
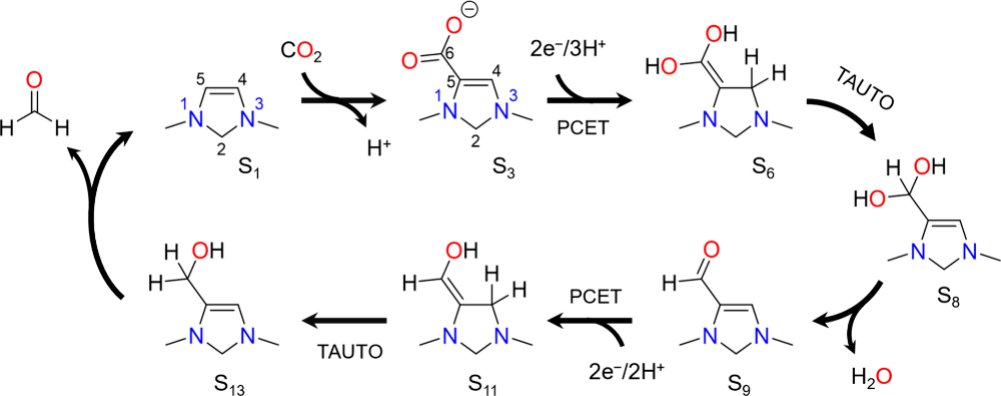
Catalytic Cycle for Reduction of CO_2_ to Formaldehyde by 1,3-Dimethyl-4-Imidazoline showing CO_2_ Activation, Proton Coupled Electron Transfer (PCET), Tautomerization (TAUTO), Dehydration, and Formaldehyde Elimination Reproduced from Ref.^8^ Copyright 2024 American Chemical Society.

Recently, we demonstrated in a computational study that an electro-organocatalyst containing a vicinal enediamine backbone is capable of electrochemically reducing CO_2_ to formaldehyde at a potential of −0.85 V vs RHE with a turnover frequency on the order of 0.1–1 s^–1^.^8^ The catalytic cycle occurs by the mechanism depicted in Scheme 1 and corresponds to the overall four-electron reduction,
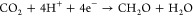


It was found that the electron-rich π system of the vicinal enediamine backbone is able to activate CO_2_ by formation of a C–C bond and then facilitate outer sphere electron transfer from an inert cathode. Unlike transition metal electrocatalysts, the electro-organocatalyst is not capable of producing undesired products such as CO and H_2_ since no mechanism exists for elimination of these species from an organic molecule. Additionally, we postulated a mechanism by which this electro-organocatalyst could be capable of forming long chain aldehydes by carrying out reductive aldol condensation to grow an alkyl chain one carbon at a time. One iteration of the chain growth process involves reductive addition of formaldehyde to a second aldehyde to produce a product aldehyde that is longer by one carbon atom,
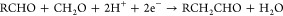


Coupled with the CO_2_ reduction cycle, the chain growth process effectively corresponds to chain growth by reductive addition of CO_2_,
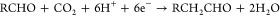
producing long chain aldehydes from nothing but CO_2_, protons, and electrons,
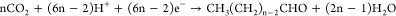


Not only are these long chain aldehydes a more valuable product than formaldehyde, but our calculations also show that in the absence of this chain growth process the electro-organocatalyst would become inactivated by buildup of the formaldehyde product to concentrations in excess of a few mmol/L. Thus, the chain growth process is actually an integral part of the reduction of CO_2_ by this electro-organocatalyst.

Formation of the C–C bond in the chain growth cycle occurs by an “umpolung” mechanism that involves reversal of polarity on one of the two carbon atoms forming the bond. Organocatalysts such as N-heterocyclic carbenes (NHCs) have been extensively studied for their ability to carry out such reactions.^9,10^ The formose reaction occurs by a similar mechanism in which glycoaldehyde functions as both the product and the organocatalyst.^11^ The formoin reaction is a juxtaposition of these two types of reactions, whereby thiazolium NHCs catalyze the benzoin condensation of formaldehyde into carbohydrates.^12^ In all three cases, the organocatalyst activates an aldehyde by attack of a nucleophilic carbon center on the carbonyl group of the aldehyde,
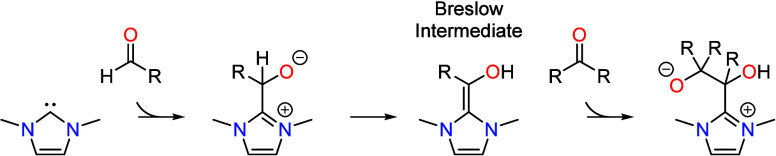


Tautomerization leads to the formation of a Breslow intermediate in which the “carbonyl” carbon is rendered nucleophilic by conjugation with electron donating groups on the organocatalyst.^13,14^ This carbon can then function as a nucleophile in an aldol addition step to an electrophilic center on a second reactant molecule in reactions such as Benzoin condensation,^15^ formoin condensation,^12^ and the Stetter reaction.^16^ While the vicinal enediamine electro-organocatalyst carries out the C–C bond formation step by a similar mechanism, it is also able to facilitate electron transfer to the resulting product leading to reductive dehydroxylation.

In the current manuscript, we computationally explore the chain growth process by an electro-organocatalyst containing a vicinal enediamine backbone. Using density functional theory, we show that it is indeed feasible as we previously hypothesized and that it operates at a comparable turnover frequency to the CO_2_ reduction cycle even at low concentrations of formaldehyde. We find that the chain growth cycle has many mechanistic similarities to the CO_2_ reduction cycle, with both being facilitated by the special catalytic properties of the vicinal enediamine motif.

## 2 Overview of the CO_2_ Reduction Cycle Catalyzed by a Vicinal Enediamine Electro-Organocatalyst

Previously, we showed that an electro-organocatalyst with a vicinal enediamine functionality is capable of electrochemically reducing CO_2_ to formaldehyde by the catalytic cycle depicted in Scheme 1.^8^ This mechanism was based on a representative electro-organocatalyst 1,3-dimethyl-4-imidazoline (DM4Im) containing the vicinal enediamine backbone. The cycle begins with CO_2_ activation occurring by an electrophilic substitution mechanism, which is enabled by the electron-rich π system of the vicinal enediamine backbone,
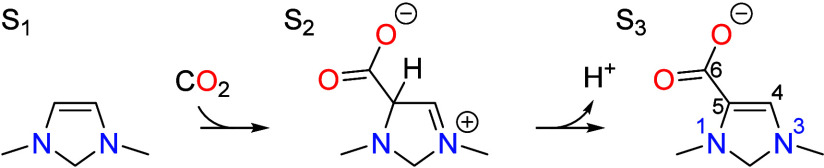


Deprotonation of the C5 position in S_2_ to form S_3_ occurs with the aid of a proton transfer mediator (PTM) that could take the form of a molecule, polymer, or surface having a p*K*_a_ close to the operating pH. Following CO_2_ activation, a sequence of proton coupled electron transfer (PCET) steps occurs that leads to a two-electron reduction of S_3_ to S_6_,
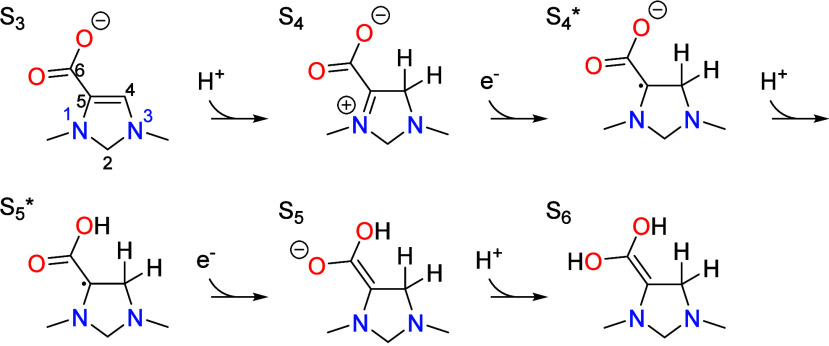


The PCET is initiated by protonation of S_3_ at the C4 position to give S_4_, with the proton being donated by the PTM. This is the rate limiting step of the catalytic cycle and has a rate that is independent of the cathode potential since it is a nonelectrochemical step. Following protonation, S_4_ readily undergoes alternating transfer of two electrons from the cathode and two protons from the PTM to form S_6_. This is then followed by tautomerization and dehydration of the resulting geminal diol to give S_9_ in which the carboxylate group formed from CO_2_ addition is transformed into an aldehyde. The aldehyde then undergoes a second PCET sequence followed by tautomerization that converts the aldehyde in S_9_ into a hydroxymethyl group in S_13_. The hydroxymethyl is finally eliminated as formaldehyde to complete the catalytic cycle and return the catalyst to the initial state S_1_.

There are several key features of the vicinal enediamine electro-organocatalyst that enable it to carry out the CO_2_ reduction cycle, which will be shown to also have importance in the chain growth cycle. The addition of CO_2_ and elimination of formaldehyde proceed through zwitterionic intermediates (e.g., S_2_) that are stabilized by favorable placement of the resulting positive charge on the N3 nitrogen atom. This property also enables facile dehydration of the geminal diol in the step S_8_ → S_9_. Likewise, placement of positive charge on the N1 nitrogen atom facilitates rapid tautomerization in steps S_6_ → S_8_ and S_11_ → S_13_ even at neutral pH.

The electron transfers involved in the two PCET sequences are favorable at modest overpotentials because the initial and final states avoid unfavorable placement of formal positive or negative charge on any carbon atom due to the unique structure of the electro-organocatalyst. Instead, positive charge is placed on one of the nitrogen atoms in the initial state (e.g., S_4_ → S_4^*^_) or negative charge is placed on an oxygen atom in the final state (e.g., S_5^*^_ → S_5_). This feature of the electro-organocatalyst is discussed further in Section 6.5 for an analogous PCET step that occurs during the chain growth cycle.

## 3 Density Functional Theory Calculations

We make use of a free energy profile to visualize the kinetics of the elementary steps and the overall chain growth cycle discussed in the previous section. Full details of the DFT calculations carried out to construct this profile are given in the Supporting Information and in ref.^8^ These calculations were performed using the Vienna Ab initio Simulation Package^17^ (VASP) along with the VASPsol extension^18,19^ that allows for implicitly modeling the electrolyte by a continuum electrostatic description. The Bayesian error estimation functional with van der Waals correlation (BEEF-vdW)^20^ was used in all calculations. This functional was chosen based on its balanced accuracy for describing a wide range of energetic quantities, ranging from molecular formation, reaction, and activation energies to cohesive energies of solids and chemisorption on solid surfaces. In addition, the BEEF-vdW functional includes dispersion interactions such as those that would occur between molecules and surfaces. While surfaces are not included in the present study, we envision combining this system with a solid proton transfer mediator such as a metal oxide surface in the future. To account for errors in the description of certain functional groups by semilocal exchange-correlation functionals such as BEEF-vdW, empirical corrections are applied to the electronic energy of CO_2_ and any molecule with a carboxylate, carbonyl, or geminal diol group.

Transition states for most steps were found by performing a roughly converged nudged elastic band calculation^21,22^ to obtain an initial guess, followed by a dimer calculation^23^ to refine the transition state. We neglect activation barriers for steps involving proton transfer between the PTM and an oxygen atom (S_15_ → S_14_, S_18_ → S_19_, S_24_ → S_25_, S_29_ → S_30_) as well as steps involving electron transfer (S_25_ → S_25^*^_, S_26^*^_→ S_26_) since the energetic barriers of such steps were found to be negligible in our previous work.^8^

As generalized gradient approximation (GGA) functionals such as BEEF-vdW are known to underestimate activation barriers for proton transfer reactions,^24^ we have also performed benchmarking calculations using hybrid DFT functionals on two of the key proton transfer steps, S_3_ → S_4_ in the CO_2_ reduction cycle and S_19_ → S_20_ in the chain growth cycle. These calculations were performed in NWChem^25^ using the PBE0^26^ and B3LYP^27^ functionals. We find that the activation barriers calculated using these hybrid functionals are no more than 0.08 eV higher than those calculated using the BEEF-vdW functional. This is due to the fact that the BEEF-vdW functional underpredicts these barriers less severely than more widely used GGA functionals such as PBE,^28^ consistent with reported results for other nonlocal van der Waals GGA functionals.^24^ Further details of the benchmarking calculations are discussed in the Supporting Information.

Transition states for steps involving proton transfer between the proton transfer mediator (PTM) and a carbon atom were computing using formic acid or formate as a model PTM. The barriers were then extrapolated to a PTM having a p*K*_a_ equal to the pH using the approach developed in our previous work on the CO_2_ reduction cycle.^8^ The extrapolation is based on an analogy with Marcus theory for electron transfer reactions.^29^ For a protonation step S_*i*_ → S_*j*_, the effective activation barrier with a PTM having a p*K*_a_ equal to the pH is given by,
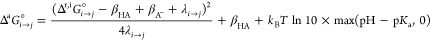
1where *λ*_*i*__→*j*_ is the reorganization energy and p*K*_a_ corresponds to the model PTM (formic acid/formate). The parameters β_HA_ and β_A^–^_ are constants that account for the free energy to form a precursor complex between the reactant or product and the PTM. The intrinsic reaction free energy is given by,
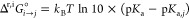
2where p*K*_a*,j*_ is the p*K*_a_ of the product S_*j*_. A similar extrapolation is used for a deprotonation step S_*i*_ → S_*j*_,
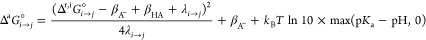
3where the intrinsic reaction free energy is given by (p*K*_a*,i*_ is the p*K*_a_ of the reactant S_*i*_),
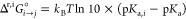
4

Further details of this approach, including the parameter values, are given in the Supporting Information.

To account for interactions between the catalytic intermediates and the electrolyte, we employ a hybrid implicit-explicit solvation method that was developed in our previous manuscript.^8^ Including certain water molecules explicitly in the DFT calculations allows for an appropriate description of strong hydrogen bonds that can form between the solute and solvating water molecules, particularly in charged intermediates and transition states. This hybrid solvation method differs from other similar methods (c.f. refs^30^ and^31^) by applying an empirical hydrogen bonding correction to account for the entropy loss associated with formation of hydrogen bonds, rather than computing this contribution explicitly from vibrational frequency calculations. Doing so avoids the difficulties and errors associated with applying the harmonic approximation to lose vibrational modes associated with hydrogen bonding interactions. The correction, *G*_W,corr_, is determined by fitting the self-solvation free energy of water to the experimental value.

Free energies of intermediates and transition states were determined by adding translational, rotational, and vibrational contributions to the electronic energy *E*_0,aq_ computed by VASP with implicit solvation,
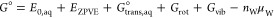
5

The last term accounts for the effective chemical potential of the *n*_W_ molecules of explicitly hydrogen bonded water,
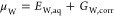
6where *E*_W,aq_ is the electronic energy of a water molecule implicitly “solvated” in the electrolyte. The number of explicit water molecules is determined based on the commonly used “variational” approach,^30^ by selecting the configuration with the lowest free energy according to eq 5.

## 4 Mechanism of the Chain Growth Catalytic Cycle

**Scheme 2:**
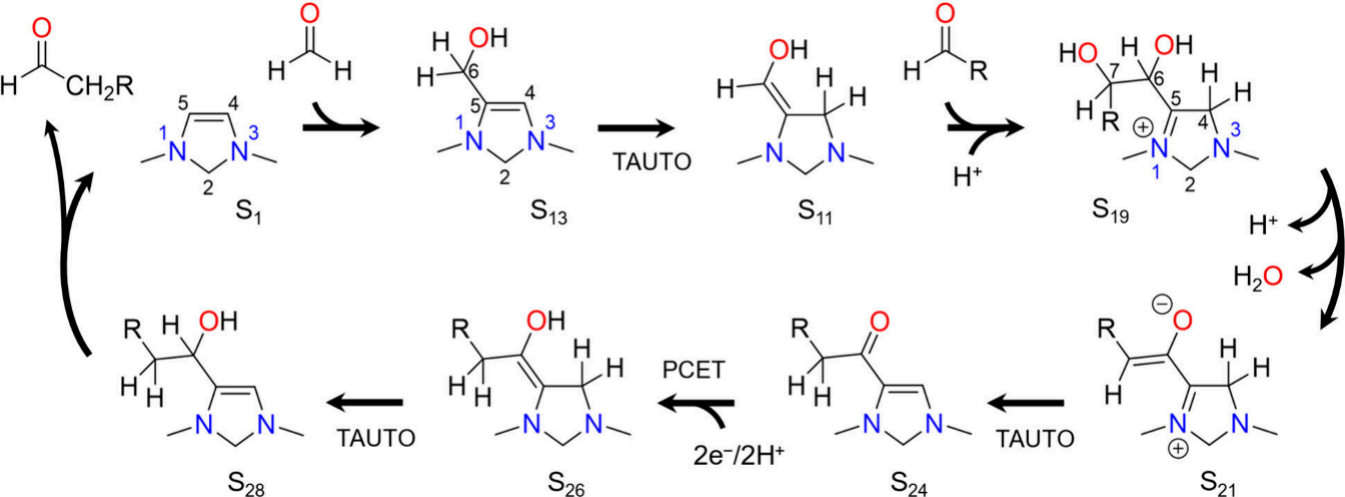
Catalytic Cycle for Aldehyde Chain Growth by 1,3-Dimethyl-4-imidazoline Showing Formaldehyde Activation, Tautomerization (TAUTO), Aldol Addition, Dehydration, Proton Coupled Electron Transfer (PCET), and Aldehyde Elimination

The chain growth catalytic cycle is depicted in Scheme 2 and is based on the same representative electro-organocatalyst 1,3-dimethyl-4-imidazoline (DM4Im) used to demonstrate the CO_2_ reduction cycle in our previous work. It can be seen that it features many of the same elementary steps as the CO_2_ reduction cycle such as tautomerization, dehydration, PCET, and aldehyde elimination. The cycle begins with the reverse of the last part of the CO_2_ reduction cycle, whereby formaldehyde is activated by the electro-organocatalyst and tautomerizes to give the enol intermediate S_11_. This intermediate is analogous to the Breslow intermediate involved in C–C bond formation catalyzed by N-heterocyclic carbene catalysts.^13,14^ Once formed, it undergoes aldol addition of an aldehyde to the C6 position to give S_19_. The C6 carbon of S_11_ is rendered nucleophilic by polarity reversal from conjugation with the lone pair on N1, enabling it to attack the electrophilic carbonyl of the aldehyde. As with aldehyde addition/elimination and CO_2_ addition, the aldol addition proceeds through a zwitterionic intermediate S_18_ in which positive charge is formally placed on the N1 nitrogen.

Intermediate S_19_ undergoes deprotonation of C6 followed dehydration of C7 (the carbonyl position of the aldehyde) to give S_21_. The deprotonation step involves proton transfer to the PTM to give S_20_, which then dehydrates by a concerted mechanism in which a proton is shuttled from the hydroxyl on C6 to the hydroxyl on C7 followed by water elimination. Intermediate S_21_ then tautomerizes to S_24_ by transfer of a proton from the C4 position to the C7 position via the cationic intermediate S_22_.

Intermediate S_24_ resembles S_9_ of the CO_2_ reduction cycle except that the hydrogen on C6 is replaced by an alkyl group. The remainder of the chain growth cycle proceeds by an analogous mechanism to the second half of the CO_2_ reduction cycle (from S_9_); S_24_ undergoes a PCET sequence to yield S_26_ which then tautomerizes to S_28_ and eliminates an aldehyde to complete the catalytic cycle.

**Scheme 3:**
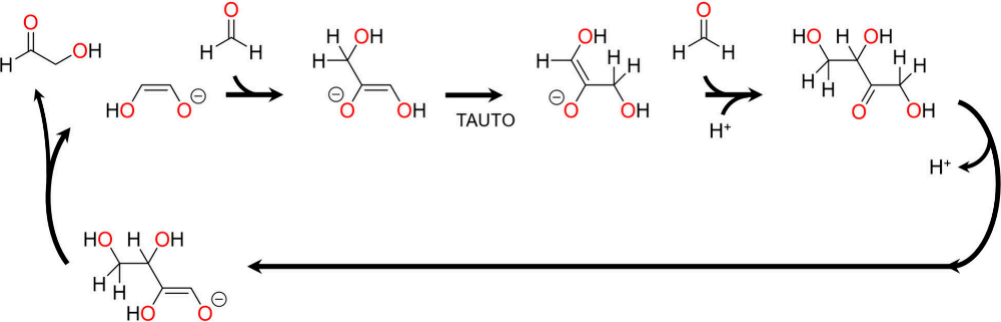
Catalytic Cycle for the Formose Reaction, Highlighting the Similarities with the Chain Growth Cycle in Scheme 2 Adapted with permission from Ref.^11^ Copyright 1959 Elsevier Ltd.

A similar mechanism for C–C bond formation occurs in the formose reaction,^11^ whereby two successive aldol additions of formaldehyde to glycolaldehyde followed by retro-aldol elimination produces a second molecule of glycoaldehyde. The reaction is autocatalytic, with glycolaldehyde acting as both a product and an organocatalyst. The catalytic activity of glycolaldehyde results from deprotonation to its enediolate form, which can then undergo aldol addition with formaldehyde. This is followed by tautomerization and aldol addition of a second molecule of formaldehyde, with the cycle completed by retro-aldol elimination of glycoaldehyde. The mechanism is depicted in Scheme 3, which has been drawn in such a way to highlight the similarities between the formose reaction and the chain growth cycle in Scheme 2. It can be seen that the vicinal enediamine electro-organocatalyst functions in an analogous way, carrying out two successive additions of formaldehyde. However, the electro-organocatalyst is also capable of facilitating dehydration and electron transfer so that acetaldehyde is formed as the product instead of glycoaldehyde.

## 5 Free Energy Profile of the Chain Growth Cycle

As detailed in our previous work,^8^ the free energy profile is constructed from the free energies of each catalytic intermediate (Δ*G*_*i*_^◦^) and transition state (Δ*G*_*i*→*j*_^‡◦^) relative to the initial state S_1_ of the catalytic cycle. A reactive intermediate S_*i*_ formed from S_1_ by a process involving protons, electrons, and any number of other reactants and products A_*k*_,
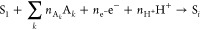
7has a relative free energy given by,
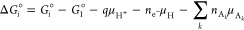
8where *G*_*i*_^◦^ and *G*_1_^◦^ are the absolute free energies computed by eq 5 of S_*i*_ and S_1_, respectively. In this expression, *q* = *n*_H^+^_ - *n*_e^–^_ is the net charge of S_*i*_. The set of reactants and products A_*k*_ consists of CO_2_, H_2_O, formic acid, and formaldehyde. Expressions for the chemical potentials μ_A_*k*__ of these reactants and products as well as the hydrogen atom and proton chemical potentials μ_H^+^_ and μ_H_ are given in the Supporting Information.

The relative free energy of the transition state for the reaction S_*i*_ → S_*j*_ is given by,
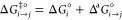
9

The activation barrier Δ^a^*G*_*i*→*j*_^◦^ is defined as the free energy difference between the transition state and the preceding intermediate S_*i*_. A special form is used for reactions involving proton transfer, which is discussed in the Supporting Information. For any other step converting S_*i*_ to S_*j*_, possibly involving one or more reactant molecule A_*k*_, the activation barrier is given by,
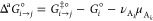
10where *G*_*i*→*j*_^‡◦^ is the absolute free energy of the transition state computed by eq 5. The stoichiometric coefficients *ν*_A_*k*__ represent the number of each molecule A_*k*_ involved in formation of the transition state.

**Figure 1:**
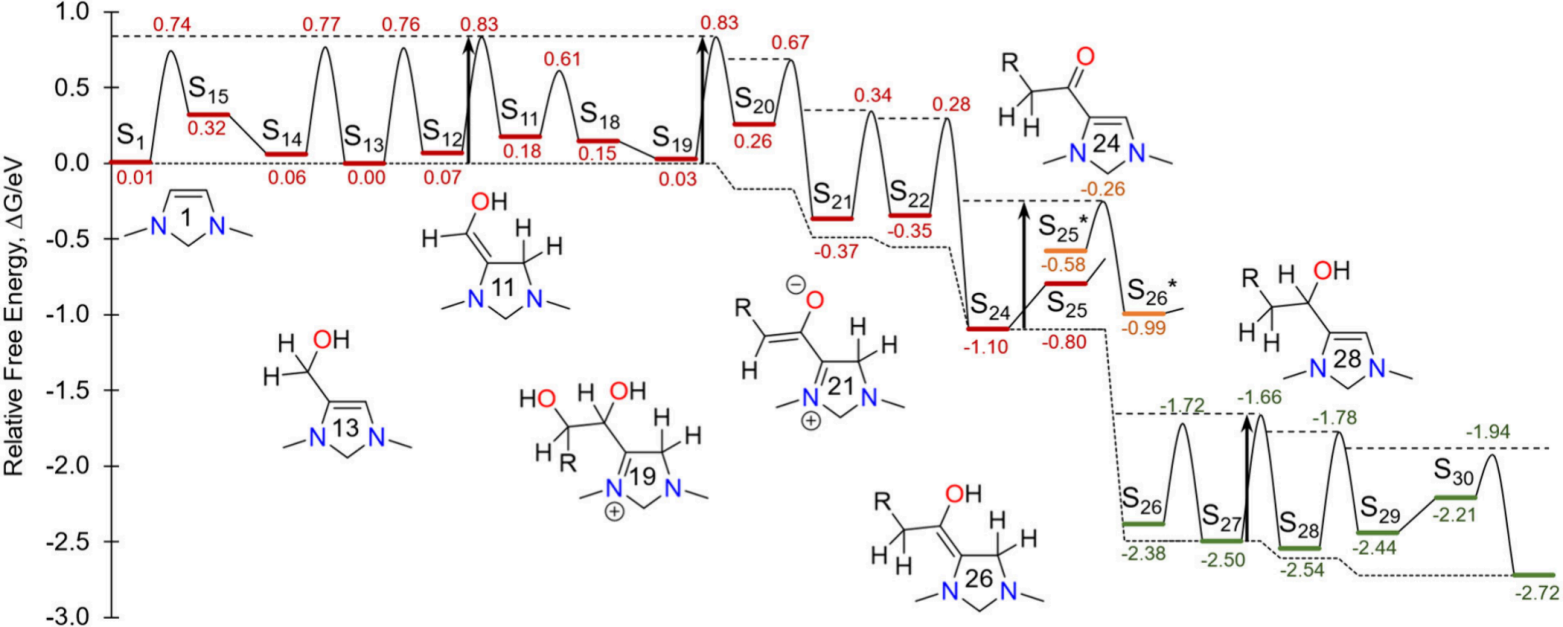
Free energy diagram of the chain growth cycle from formaldehyde to acetaldehyde. The relative free energy of each intermediate and transition state is labled in eV. The electron transfer steps are represented as vertical steps in the diagram (S_25_ → S_25^*^_, S_26^*^_ → S_26_). The upper dashed line indicates the “surface” in the “waterfall” analogy for interpreting the diagram, while the lower dashed line indicates the resting free energy along the reaction path. The four vertical arrows indicate the global barriers associated with the formal rate limiting steps S_12_ → S_11_, S_19_ → S_20_, S_25^*^_ → S_26^*^_, and S_27_ → S_28_. Free energies are computed at 80 °C, the catalytically optimal pH of 6.7, the limiting potential of −0.89 V vs RHE, and the limiting formaldehyde concentration of log[CH_2_O] = −2.4.

The free energy profile for the chain growth cycle is presented in Figure 1 for the representative electro-organocatalyst 1,3-dimethyl-4-imidazoline (DM4Im). This molecule was chosen as a model catalyst in our previous work^8^ since it is one of the simplest structures containing the vicinal enediamine backbone. The profile corresponds to a temperature of 80 °C, a pH of 6.7, an electrode potential of −0.89 V vs RHE, and a formaldehyde concentration of 4.3 mmol/L (log[CH_2_O]=–2.4). The temperature was chosen to represent an upper limit of the operating temperature for an anion exchange membrane electrolyzer while the other operating conditions were chosen to maximize the TOF of the chain growth cycle as discussed in Section 7.

As we discussed in our previous work,^8^ the free energy diagram can be related to the TOF of the catalytic cycle by the “waterfall analogy”. To see this, one visualizes water cascading from left to right down the free energy profile and pooling up behind all of the barriers corresponding to transition states. The surface of the water behind all of the barriers is indicated by the upper dashed line on the diagram and corresponds to the transition state free energies of all irreversible steps. Likewise, all of the quasi-equilibrated transition states are below this line, i.e. “under water”. The lower dashed line is identical to the upper dashed line shifted down by an amount equal to the *global barrier* for the catalytic cycle Δ*G*^‡^. We refer to this lower dashed line as the *resting free energy profile*, while we refer to the free energy profile comprised from the standard free energies of the intermediates and transition states as the *standard free energy profile*. The former depicts the free energies of the states at the steady state concentrations while the latter depicts the free energies of the states at the standard concentration of 1 mol/L. Note that the resting free energy profile is monotonically decreasing, consistent with the second law of thermodynamics; thus, the irreversibility of each step can be characterized by the drop in the resting free energy profile associated with that step. Graphically, the global barrier is the minimum amount one must shift down the resting free energy profile so that it lies below the standard free energy profile. Kinetically, it is related to the TOF of the catalytic cycle by,
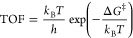


Any state lying on the resting free energy profile is a *global resting state* of the catalytic cycle and will represent a dominant state of the catalyst at steady state. In general, there is only one global resting state but under special conditions there can be two or more as is the case here.

Using the waterfall analogy, one can also identify the rate limiting steps of the catalytic cycle. First, we define the *resting state* associated with an irreversible step as the lowest free energy state to its left (or “upstream”) on the free energy diagram. A *formal rate limiting step* is an irreversible step for which the associated resting state is also a *global resting state* – in other words, the standard free energy of the resting state lies on the resting free energy profile. Looking at the free energy diagram for the chain growth cycle in Figure 1, one can see that there are four formal rate limiting steps, S_12_ → S_11_, S_19_ → S_20_, S_25^*^_ → S_26^*^_, and S_27_ → S_28_. A global barrier of 0.83 eV is associated with these four steps, corresponding to a turnover frequency of 9.3 s^–1^. In addition to these, there are three *partially rate limiting steps*, S_14_ → S_13_, S_13_ → S_12_, and S_28_ → S_29_, that have total barriers within ln 10 × *k*_B_*T* (0.07 eV) of the global barrier. The formal rate limiting steps are associated with four global resting states, S_0_, S_13_, S_24_, and S_27_. Five other states (S_1_, S_14_, S_12_, S_19_, and S_29_) are close to being global resting states, lying within ln 10 × *k*_B_*T* (0.07 eV) of the resting free energy profile. The four global resting states along with the five states that are nearly global resting states would all be expected to have appreciable steady state concentrations.

It is no coincidence that multiple rate limiting steps exist; this occurs because the free energy profile has been calculated at the optimal conditions for operating the catalytic cycle. In particular, the precise values chosen for the three operating conditions (pH, electrode potential, and formaldehyde concentration) lead to the occurrence of the three additional rate limiting steps. To aid in understanding why this occurs, we define the *total barrier* for an irreversible step in the catalytic cycle as the difference in free energy between its transition state and its resting state. For a formal rate limiting step, this latter state will be a global resting state and its total barrier will be equal to the global barrier. In Section 7, we discuss how the total barriers of various steps are affected by pH, electrode potential, and formaldehyde concentration and how this leads to changes in the formal rate limiting step under different conditions.

## 6 Elementary Steps in the Chain Growth Cycle

We now discuss the energetic and mechanistic details of the individual elementary steps in the chain growth cycle. The energies discussed will be those corresponding to the optimal conditions for operating the isolated chain growth cycle (pH = 6.7, U = −0.89 V vs RHE, log[CH_2_O] = −2.4), which is discussed in detail in Section 7. Most of these energies will be somewhat different at the optimal conditions for coupling with the CO_2_ reduction cycle (pH = 7.8, U = −0.85 V vs RHE, log[CH_2_O] = −3.1), which is discussed in Section 8.

### 6.1 Formaldehyde Activation

The initial state of the organocatalyst is in equilibrium between the active state (S_1_) and an inactive state (S_0_) in which the C5 position is protonated,
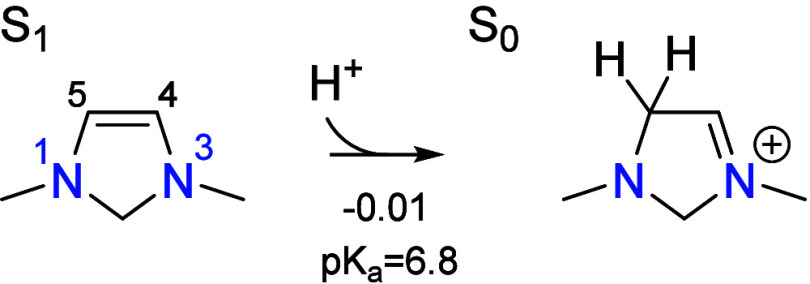


The calculated p*K*_a_ of S_0_ is 6.8 so that the protonated state is slightly favored by −0.01 eV at the optimal pH for the isolated chain growth cycle of 6.7. In contrast, the deprotonated state is thermodynamically favored at the optimal pH (7.8) for coupling the chain growth cycle with the CO_2_ reduction cycle.

The catalytic cycle begins from the deprotonated state S_1_ by activation of formaldehyde, which occurs by electrophilic substitution at the C5 position in S_1_ to give S_13_. This occurs by the reverse of the formaldehyde elimination mechanism in the CO_2_ reduction cycle and is similar to how S_1_ activates CO_2_. Formaldehyde addition occurs in three elementary steps, the first being electrophilic addition to the C=C π bond at the C5 position to give the zwitterionic intermediate S_15_,
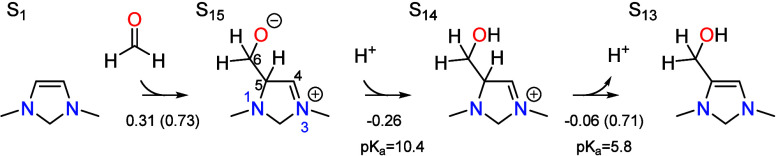


In this and other reaction schemes, the reaction free energies and activation barriers are indicated below each reaction arrow in eV, with the activation barriers enclosed by parentheses. The activation barrier for the electrophilic addition step is 0.73 eV with respect to S_1_ while the total barrier with respect to the global resting state S_0_ is 0.74 eV. At the limiting formaldehyde concentration (log[CH_2_O] = −2.4), this step is formally reversible since the transition state is lower in free energy than the transition states of two subsequent steps, S_12_ → S_11_ and S_19_ → S_20_. The p*K*_a_ associated with protonating S_15_ on the oxygen is 10.4 which readily occurs at the optimal pH of 6.7 to give S_14_. In our previous study on the CO_2_ reduction cycle,^8^ it was found that proton transfer steps to and from oxygen atoms do not have a transition state on the potential energy surface. The proton transfer occurs spontaneously in the energetically favorable direction when the proton transfer mediator (PTM) approaches the intermediate. Therefore, the barrier for this step is purely entropic and assumed to be negligible.

**Figure 2:**
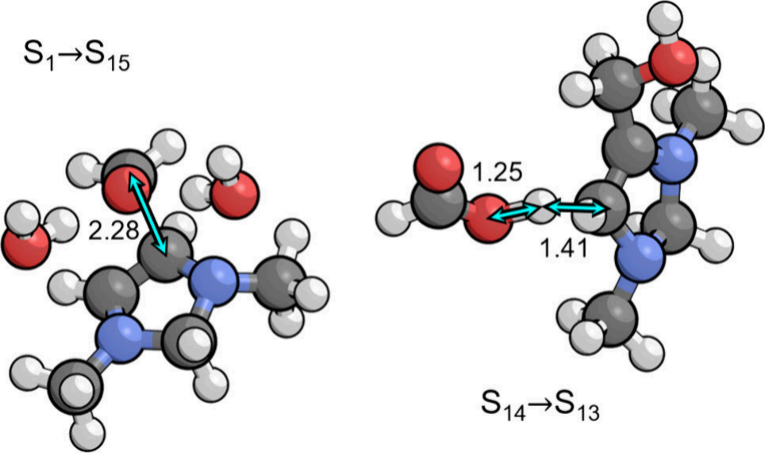
Transition states for formaldehyde addition to S_1_ and deprotonation of S_14_ by the PTM (formate) involved in formaldehyde activation (S_1_ → S_15_ → S_14_ → S_13_). Relevant bond distances are labeled in Å.

As shown in Figure 2, the transition state for S_1_ → S_15_ involves rehybridization of both carbon atoms forming the new C–C bond from sp^2^ to sp^3^, with a C–C bond distance of 2.28 Å. This is associated with an energy barrier of only 0.17 eV; however, the entropic penalty for bringing formaldehyde from the bulk electrolyte raises the free energy barrier to 0.73 eV with respect to S_1_. The additional thermodynamic barrier of 0.01 eV to deprotonate the resting state S_0_ leads to a total barrier of 0.74 eV. Both formaldehyde addition intermediates S_15_ and S_14_ are stabilized by placement of the formal positive charge on N3, making the electron-rich π system in S_1_ exceptionally nucleophilic. As a result, the cationic S_14_ is only 0.05 eV higher in free energy than the neutral S_1_. This is the same property that allows S_1_ to activate CO_2_ in the CO_2_ reduction cycle.

Once S_14_ is formed, it deprotonates at the C5 position to complete the formaldehyde addition process. The proton is transferred to the basic form of the PTM to give intermediate S_13_, which involves rehybridization of C5 from sp^3^ to sp^2^. This leads to an activation barrier of 0.71 eV and a total barrier of 0.77 eV with respect to the local resting state S_0_, making the step partially rate limiting.

Comparing to Scheme 3, it can be seen that this mechanism for formaldehyde activation is analogous to the mechanism by which formaldehyde addition occurs to the enediolate form of glycoaldehyde during the formose reaction.^11^ In the formose reaction, the α carbon of the enediolate functions as the nucleophile during this step. The enediolate is expected to be more nucleophilic than the vicinal enediamine S_1_ but requires a significantly higher pH to exist in the deprotonated state (>14 for the enediolate vs ∼7 for S_1_). As such, this step should be intrinsically slower than the initial step of the formose reaction but does not require high pH conditions that are incompatible with the presence of CO_2_.

### 6.2 Tautomerization

The chain growth cycle contains three tautomerization steps in which a proton is transferred between two carbon atoms. All three occur by a similar mechanism through a cationic intermediate in which the formal positive charge is placed on N1. The p*K*_a_ values of the carbon atoms being protonated range from 5.7 to 8.3 so that the intermediates readily form at neutral pH conditions. The first tautomerization converts S_13_ to S_11_ and is the reverse of the final tautomerization in the CO_2_ reduction cycle,
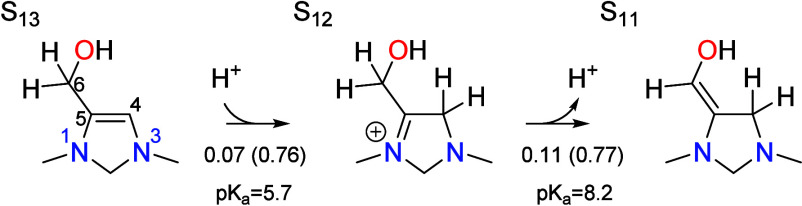


The intermediate S_12_ forms by protonation of the C4 position, having a p*K*_a_ of 5.7 so it is slightly uphill in free energy at the optimal pH of 6.7. As with other proton transfer steps, the proton is donated from the acidic form of the PTM and involves a barrier of 0.76 eV with respect to the resting state S_13_, making the step partially rate limiting. Intermediate S_12_ then deprotonates from the C6 position to give S_11_. The proton has a p*K*_a_ of 8.2 so that the deprotonation step is also uphill in free energy at the optimal pH. The deprotonation step is formally rate limiting, having a total barrier equal to the global barrier of 0.83 eV with respect to the resting state S_13_.

**Figure 3:**
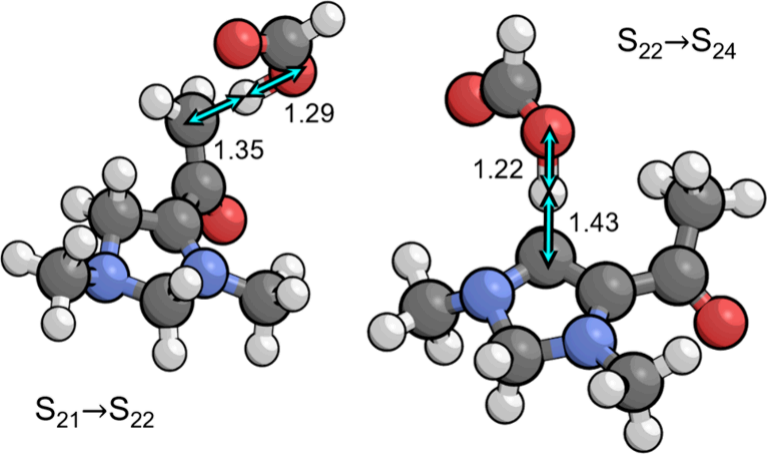
Transition states for protonation of the C7 position and deprotonation of the C4 position by the PTM (formic acid and formate, repsectively) as occur during tautomerization (S_21_ → S_22_ → S_24_). Relevant bond distances are labeled in Å.

The second tautomerization step occurs following dehydration and involves the transfer of a proton from the C4 position to the C7 position in S_21_ to yield S_24_,
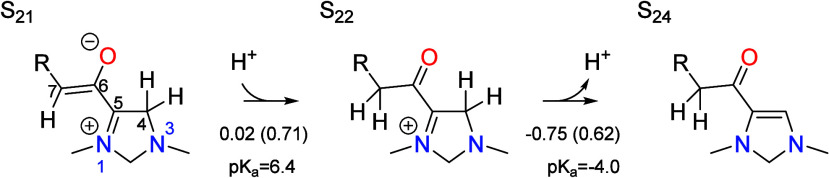


Protonation at C7 to give the S_22_ intermediate is associated with a p*K*_a_ of 6.4 and thus is only slightly higher in free energy than the resting state S_21_ at the optimal pH of 6.7. Deprotonation of S_22_ at C4 yields S_24_ and is associated with a p*K*_a_ of −4.0, making it highly favorable at the optimal pH. The unusually low p*K*_a_ value arises from stabilization in S_24_ due to conjugation of the lone pair on N3 with the carbonyl group. A similar conjugated interaction was observed in the product of the dehydration step in the CO_2_ reduction cycle, making this step highly irreversible also. The total barriers for the protonation and deprotonation steps are 0.71 and 0.65 eV, significantly lower than the global barrier of 0.83 eV; thus, neither step is rate limiting. The protonation step is slightly irreversible at the optimal pH, while the deprotonation step is highly irreversible. Together, both steps account for 22% of the irreversibility of the catalytic cycle. Both transition states are depicted in Figure 3, where it can be seen that the carbon positions involved in the proton transfer are rehybridizing between sp^2^ and sp^3^, while the proton is in transit between the PTM and the carbon atom.

The final tautomerization step is analogous to step S_11_ → S_12_ → S_13_ in the CO_2_ reduction cycle, or the reverse of this process at the beginning of the chain grown cycle. A proton is transferred from C4 to C6 and is the last process that occurs before the aldehyde is eliminated to complete the catalytic cycle,
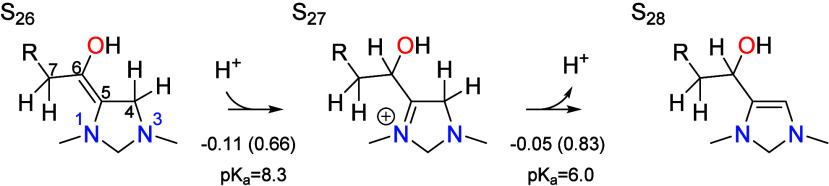


Protonation of C6 leads to the intermediate S_27_ with an associated p*K*_a_ of 8.3, while subsequent deprotonation of C4 yields S_28_ with an associated p*K*_a_ of 6.0. The protonation step is quasi-equilibrated, having an activation barrier of 0.66 eV, while the deprotonation step is formally rate limiting with a total barrier equal to the global barrier of 0.83 eV.

### 6.3 Aldol Addition

**Figure 4:**
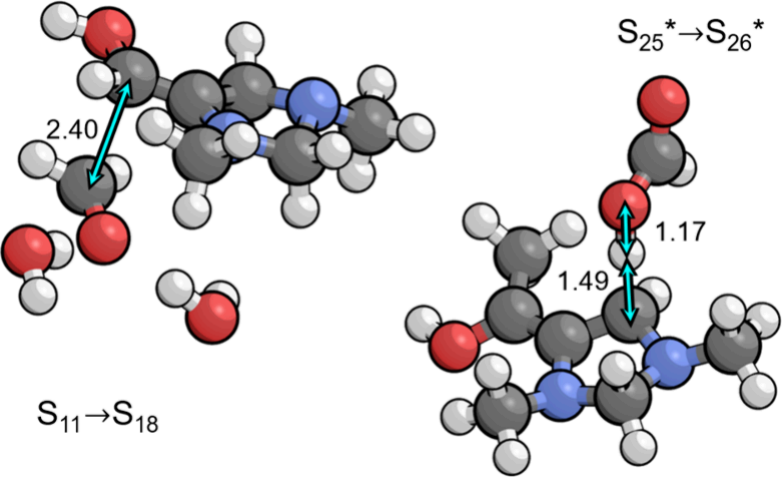
(left) Precursor complex for aldol addition to the C6 position (S_11_ → S_18_). (right) Transition state for the proton transfer step to the C4 position (S_25^*^_ → S_26^*^_) by the PTM (formic acid) that occurs during the PCET sequence (S_24_ → S_25_ → S_25^*^_ → S_26^*^_ → S_26_). Relevant bond distances are labeled in Å.

The aldol addition step results in formation of the C–C bond and occurs immediately after tautomerization of S_13_ to S_11_. Conjugation of the enolic C=C double bond in S_11_ with the lone pair on N1 reverses the polarity of the C6 position to allow the normally electrophilic carbon to function as a nucleophile instead. This enables electrophilic addition of the aldehyde at this position to give the zwitterionic intermediate S_18_,
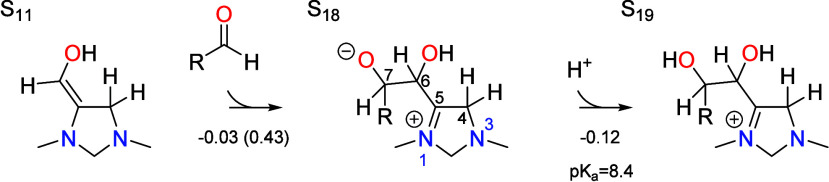


An initial precursor state depicted in Figure 4 was found in which the formaldehyde appears to form a charge transfer complex with S_11_, having an interaction energy of −0.12 eV below the energy of separated S_11_ and formaldehyde. The C–C distance is fairly short in this complex at 2.40 Å, well below the van der Waals distance of 3.40 Å. The energy of the nudged elastic band calculation between the initial and final states decreased monotonically and did not indicate any transition state, although the initial state did not spontaneously optimize to the final state. For this reason, we were unable to find a transitions state and therefore assumed that it lies very close to the initial state. We therefore used the initial precursor complex to calculate the free energy barrier for this step. Although the precursor complex is lower in energy than separated formaldehyde and S_11_, the entropy penalty for transferring the formaldehyde from the bulk electrolyte makes the free energy barrier 0.43 eV. The total barrier is 0.61 eV with respect to the resting state S_13_. The C–C bond formation step is then followed by protonation of the oxygen in the zwitterionic S_18_ intermediate to give S_19_, with an associated p*K*_a_ of 8.4 making it favorable at the optimal pH of 6.7.

The polarity reversal of C6 that enables aldol addition is a key feature of how NHCs carry out “umpolung” chemistry in reactions like benzoin condensation and the Stetter reaction.^9^ Intermediate S_11_ is analogous to the Breslow intermediate that is formed in these reactions, whereby conjugation of the C=C double bond with one or more nitrogen atoms in the NHC engenders nucleophilicity to the otherwise electrophilic carbon.^13,14^ This step is fundamentally identical to the first C–C bond formation reaction in the formoin reaction, in which formaldehyde is activated and rendered nucleophilic by addition to a thiazolium salt and subsequently undergoes aldol addition with a second formaldehyde.^12^

The aldol addition step is also analogous to the second C–C bond formation step that occurs in the formose reaction between formaldehyde and the enediolate form of dihydroxyacetone.^11^ Similar to the function of NHCs, conjugation with O^–^ makes the C=C double bond in the enediolate nucleophilic, allowing it to attack the electrophilic carbon of formaldehyde. As was the case when comparing formaldehyde activation by S_1_ to formaldehyde addition to glycoaldehyde during the formose reaction, the enediolate form of dihydroxyacetone is expected to be more nucleophilic than S_11_ while requiring a higher pH to exist in the deprotonated form (>14 for the enolate vs 8.2 for S_11_). Thus, addition to S_11_ should be intrinsically slower than the analogous step during the formose reaction but can occur at a significantly lower pH that is compatible with the presence of CO_2_.

### 6.4 Dehydration

**Figure 5:**
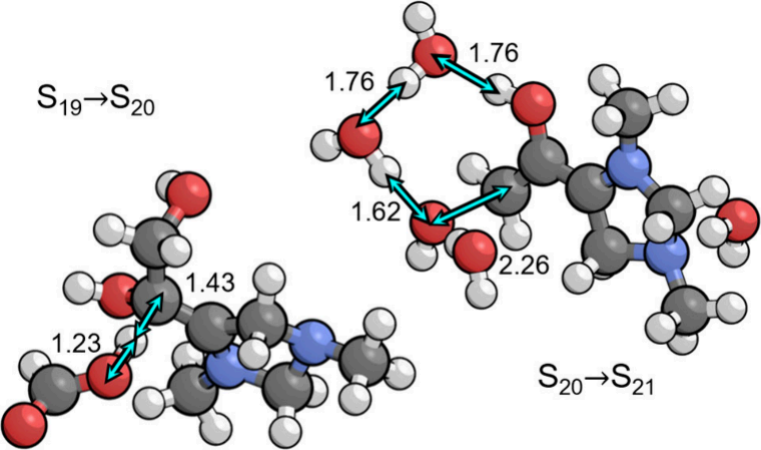
Transition states for deprotonation of the C6 position (S_19_ → S_20_) by the PTM (formate) and dehydration of the C7 position (S_20_ → S_21_) by a concerted proton shuttling mechanism. Relevant bond distances are labeled in Å.

Dehydration of the aldol addition product at the C7 position yields the zwitterionic intermediate S_21_. This process occurs in two steps,
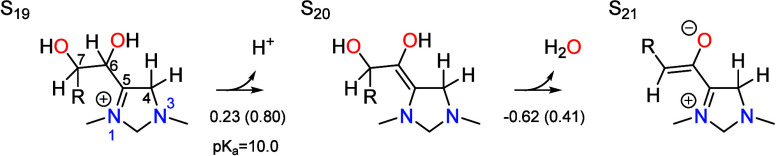


First, the C6 position of S_19_ is deprotonated to give the S_20_ intermediate. The transition state, depicted in Figure 5, is similar to those of other proton transfer reactions between carbon atoms and the PTM, particularly S_12_ → S_11_ and S_26_ → S_27_. The associated p*K*_a_ is 10.0, so the free energy is uphill by 0.23 eV at the optimal pH of 6.7. The total barrier is 0.83 eV with respect to the resting state S_13_, making this step formally rate limiting and irreversible.

Following deprotonation, S_20_ undergoes water elimination via a transition state in which the C–OH bond at the C7 position elongates to 2.26 Å, as seen in Figure 5. The oxygen in the hydroxide leaving group is strongly hydrogen bonded to three explicit water molecules, lowering the free energy of the transition state by 0.79 eV compared to the case with only implicit solvation. One of these water molecules is bridged by a fourth explicit water molecule to create an uninterrupted hydrogen bonding network between the OH group at the C6 position and the hydroxide. This provides additional stabilization compared to the structure where the hydrogen bonding bridge is not formed. The intrinsic barrier with respect to S_20_ is 0.41 eV, giving a total barrier of 0.67 eV with respect to the resting state S_13_. Consequently, this step is not rate limiting, although it is highly irreversible. Together, both steps involved in dehydration account for 18% of the total irreversibility of the cycle. As in several other steps, the zwitterionic dehydration product S_21_ is stabilized by formal placement of the positive charge on N1.

### 6.5 PCET Sequence

The PCET sequence follows the second tautomerization step S_21_ → S_24_ and is analogous to the second PCET sequence in the CO_2_ reduction cycle. It involves the transfer of two electrons and two protons to S_24_, yielding S_26_,
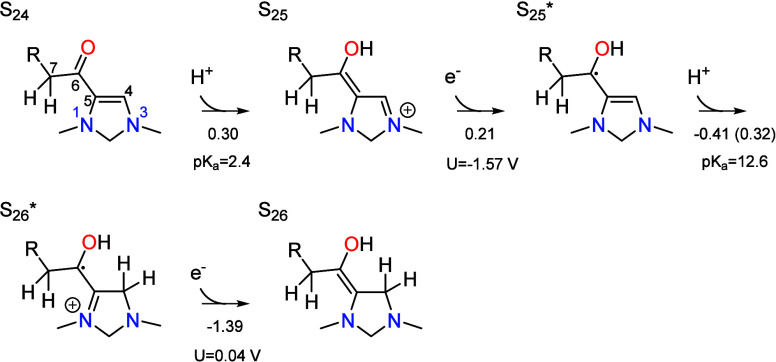


The process commences with protonation of the oxygen in S_24_, which is associated with a p*K*_a_ of 2.4. The p*K*_a_ is significantly higher than for a typical carbonyl oxygen due to delocalization of the positive charge onto N3 via conjugation. As with S_15_ → S_14_, no transition state was explicitly calculated since we have previously found that the transition state is purely entropic for proton transfer to or from oxygen. The proton transfer is then followed by an electron transfer from a chemically inert cathode to give the radical intermediate S_25^*^_. This electron transfer requires a potential of −1.57 V vs SHE to be thermodynamically favorable, making the process uphill in free energy at the operating potential of −1.33 V vs SHE. Nonetheless, the thermodynamic potential is comparatively low for electron transfer to a singlet organic molecule due to the unique structure of the electro-organocatalyst. Specifically, the unpaired electron is delocalized over the six atoms forming the conjugated π system. The reduction potential of the combined PCET step S_24_ → S_25^*^_ is −1.40 V vs RHE, leading to an overall thermodynamic barrier for this process of 0.52 eV at the limiting potential of −0.89 V vs RHE.

After S_25^*^_ is formed, the C4 position protonates to give S_26^*^_ via the transition state depicted in Figure 4. The associated p*K*_a_ is 12.6 making the proton transfer highly favorable at the optimal pH of 6.7. This leads to a relatively low barrier of 0.32 eV with respect to S_25^*^_, giving a total barrier of 0.83 eV with respect to the local resting state S_24_. This makes the step formally rate limiting at the limiting potential of −0.89 V vs RHE and is in fact the condition that defines the limiting potential as discussed in Section 7.2. The protonated intermediate S_26^*^_ then undergoes a highly irreversible electron transfer accounting for 51% of the total irreversibility of the cycle.

### 6.6 Aldehyde Elimination

**Figure 6:**
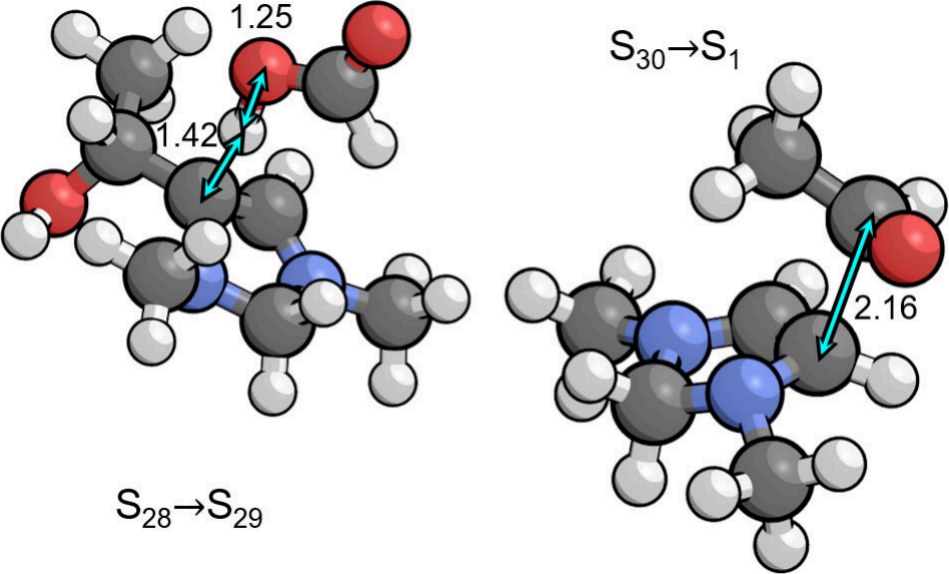
Transition states for protonation of the C5 position by the PTM (formic acid) and subsequent elimination of acetaldehyde. Relevant bond distances are labeled in Å.

Aldehyde elimination follows the third tautomerization step S_26_ → S_28_ to complete the catalytic cycle and return the catalyst to the initial state S_1_. The process follows a mechanism analogous to formaldehyde elimination in the CO_2_ reduction cycle, or equivalently the reverse of formaldehyde addition at the beginning of the chain growth cycle,
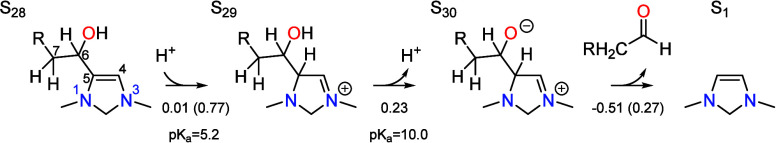


It begins with protonation of the C5 position in S_28_ to give S_29_ by the transition state depicted in Figure 6. This step has a barrier of 0.77 eV with respect to the local resting state S_28_, making it partially rate limiting. As will be seen in Section 7.1, the optimal pH of 6.7 is actually defined by the pH where this step has the same barrier as the preceding tautomerization step S_27_ → S_28_. The associated p*K*_a_ is 5.2, making the step slightly uphill in free energy at the optimal pH. The protonation step is then followed by deprotonation of the hydroxyl on C6 to form the zwitterionic intermediate S_30_. This step has a p*K*_a_ of 10.0 so that it is fairly uphill in free energy. As with the other steps involving proton transfer to or from oxygen, we assume there is no energetic transition state for this step; only an entropic transition state that is considered not to be kinetically relevant.

The final step involves elimination of the product aldehyde from S_30_. This is enabled by the charge separation in the zwitterionic intermediate, whereby a lone pair on the deprotonated oxygen makes the aldehyde an excellent electrophilic leaving group. Likewise, the iminium C=N^+^ bond between N1 and C4 is able to accept the electrons from the dissociating C–C bond. These effects combine to give a low intrinsic barrier of 0.27 eV with respect to S_30_ and a total barrier of 0.60 eV with respect to the local resting state S_28_.

## 7 Kinetic Dependence of the Chain Growth Cycle

In the previous sections, we discussed the mechanism and free energy profile of the chain growth cycle calculated at the optimal pH of 6.7, the limiting potential of −0.89 V vs RHE, and the limiting formaldehyde concentration of log[CH_2_O] = −2.4. In this section, we explore how the barriers of different steps in the cycle depend on these conditions and ultimately how these conditions are determined by kinetic trade-offs between various steps.

### 7.1 Effect of pH on the Free Energy Profile

**Figure 7:**
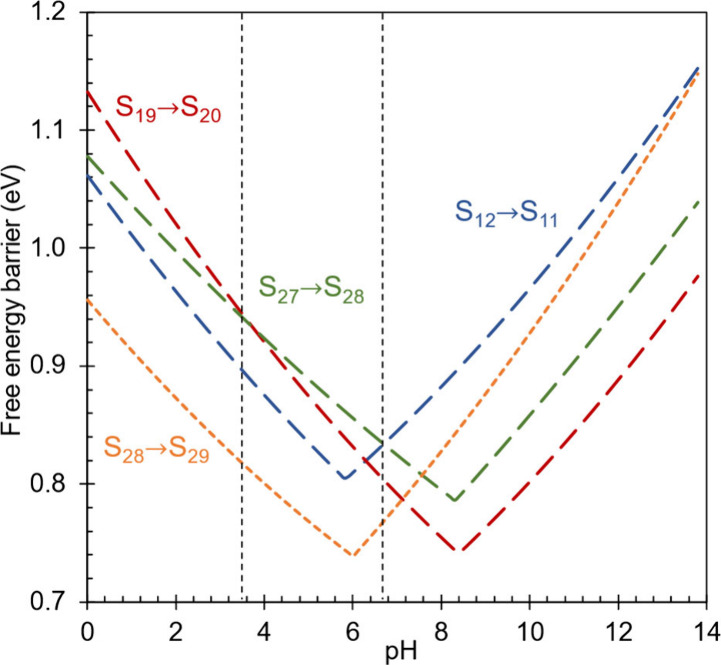
Total barriers for possible rate limiting steps with respect to electrolyte pH, calculated using [CH_2_O] = 1 mol/L. Protonation steps (S_28_ → S_29_) are indicated by short dashed lines and deprotonation steps (S_12_ → S_11_, S_19_ → S_20_, and S_27_ → S_28_) are indicated by long dashed lines. The dashed vertical lines indicate the boundaries between the kinetic regimes where different steps are formally rate limiting. The optimal pH (6.7) corresponds to the boundary where the formal rate limiting step shifts from S_27_ → S_28_ to S_28_ → S_29_.

The total barriers (defined in Section 5) for several steps have been plotted in Figure 7 as a function of the electrolyte pH, where it can be seen that all of them exhibit kinks at particular pH values where the resting state for that step changes from one state to another. By definition, the global barrier for a given pH is equal to the highest total barrier at that pH, and the corresponding step is the formal rate limiting step. Across the entire pH range, one can identify three steps that are formally rate limiting in different regions: S_19_ → S_20_ at pH values below 3.7, S_27_ → S_28_ at pH values between 3.7 and 6.7, and S_12_ → S_11_ at pH values above 6.7.

The lowest global barrier occurs at the optimal pH of 6.7 where the formal rate limiting step changes from S_27_ → S_28_ to S_12_ → S_11_. As will be discussed in more detail below, both steps are deprotonation steps but S_12_ → S_11_ proceeds from an unprotonated resting state (S_13_) while S_27_ → S_28_ proceeds from a protonated resting state (S_27_). Therefore, the total barrier for S_12_ → S_11_ increases with pH due to the dependence of the S_13_ → S_12_ quasi-equilibrium on pH, while the total barrier for S_27_ → S_28_ decreases with pH. The point where the total barriers of these two steps cross defines the optimal pH of 6.7.

To better understand the reason underlying the changes in rate limiting at pH values of 3.7 and 6.7, it is first necessary to discuss the somewhat counterintuitive behavior exhibited by the total barriers of protonation steps at low pH and of deprotonation steps at high pH. Naively, one might expect the total barrier of a protonation step to continuously decrease as pH decreases. This is true as long as the resting state of the protonation step is the preceding unprotonated species. However, when the pH is low enough that the resting state shifts to an earlier protonated species the trend in the total barrier reverses and it starts to increase with decreasing pH. This occurs because the transition state for the protonation step is actually “less” protonated than the resting state since the resting state is fully protonated while the transition state is only partially protonated (as the proton is in transit). Therefore, the free energy of the resting state decreases faster than the free energy of the transition state as the pH decreases, leading to an increase in the total barrier. This behavior is seen for S_28_ → S_29_ when the pH is lower than 6.0. When the pH is above this value, the resting state is the unprotonated state S_28_ so that the total barrier decreases as pH decreases. Below this pH though, the resting state switches to the protonated state S_27_ so that the total barrier now starts to increase as pH decreases further.

An analogous argument can be made for the increase in total barrier of a deprotonation step at sufficiently high pH. As pH increases, all three deprotonation steps depicted in Figure 7 initially exhibit decreasing barriers because the corresponding resting states are protonated: S_27_ for S_27_ → S_28_, S_19_ for S_19_ → S_20_, and S_14_ for S_12_ → S_11_ (although the free energy of S_12_ is only 0.01 eV higher than S_14_). As pH increases, the resting state for S_12_ → S_11_ switches from the protonated S_14_ to the unprotonated S_13_ at a pH of 5.8. Further increasing the pH to 8.3 switches the resting state for S_27_ → S_28_ from the protonated S_27_ to the unprotonated S_26_. Finally, increasing the pH past 8.4 switches the resting state for S_19_ → S_20_ from the protonated S_19_ to the unprotonated S_18_. Once the resting state switches to an unprotonated state, the total barrier begins to increase with pH rather than decreasing since the transition state of the deprotonation step is now “more” protonated than the fully unprotonated resting state.

The pH dependence of the total barriers of all other steps in the catalytic cycle are reported in Figure S3 in the Supporting Information. All of the proton transfer steps exhibit the same “inverted volcano” behavior where the “peak” is associated with a change from a protonated to an unprotonated resting state. The steps that do not involve proton transfer also exhibit a slope discontinuity in the pH dependence, whereby the total barrier decreases with pH at low pH before becoming independent of pH at higher pH. This is also caused by a change from a protonated to an unprotonated local resting state, but the shape is different because the reaction free energy of the step itself (and thus the intrinsic barrier) does not depend on pH since the step does not directly involve proton transfer. All of these steps proceed directly from an unprotonated state; therefore, when this state happens to be the resting state for the reaction step, the total barrier is equal to the intrinsic barrier of the step, which is independent of pH. At low pH when the resting state switches to a protonated state earlier in the cycle, there is an additional free energy penalty associated with deprotonating the resting state before the reaction step can occur. This thermodynamic penalty increases as pH decreases leading to an increase in the total barrier of ln 10 *k*_B_*T* ≈ 0.07 eV per unit decrease in pH.

We can now better understand the change in the formal rate limiting step from S_27_ → S_28_ to S_12_ → S_11_ at a pH of 6.7. Despite both being deprotonation steps, S_12_ → S_11_ proceeds from the unprotonated resting state S_13_ so that the total barrier increases with pH, while S_27_ → S_28_ proceeds from the protonated resting state S_27_ so that the total barrier decreases with increasing pH. Therefore, S_27_ → S_28_ has a higher total barrier below a pH of 6.7 while S_12_ → S_11_ has a higher total barrier above this pH.

The reason for the change in formal rate limiting step from S_19_ → S_20_ to S_27_ → S_28_ at a pH of 3.7 is more subtle. Since both steps involve deprotonation proceeding from an unprotonated resting state, the total barriers of both steps increase as pH decreases; however, the barrier for S_19_ → S_20_ increases faster than the barrier for S_27_ → S_28_. This occurs because S_19_ → S_20_ is thermodynamically less favorable than S_27_ → S_28_ and thus has a transition state that is closer to the product state. Therefore, the barrier of S_19_ → S_20_ is more sensitive to changes in the reaction free energy caused by changes in pH. As the pH decreases, the total barrier for S_19_ → S_20_ eventually becomes higher than the total barrier for S_27_ → S_28_ at a pH of 3.7, making the former step rate limiting at pH values lower than this.

### 7.2 Effect of the Electrode Potential on the Free Energy Profile

**Figure 8:**
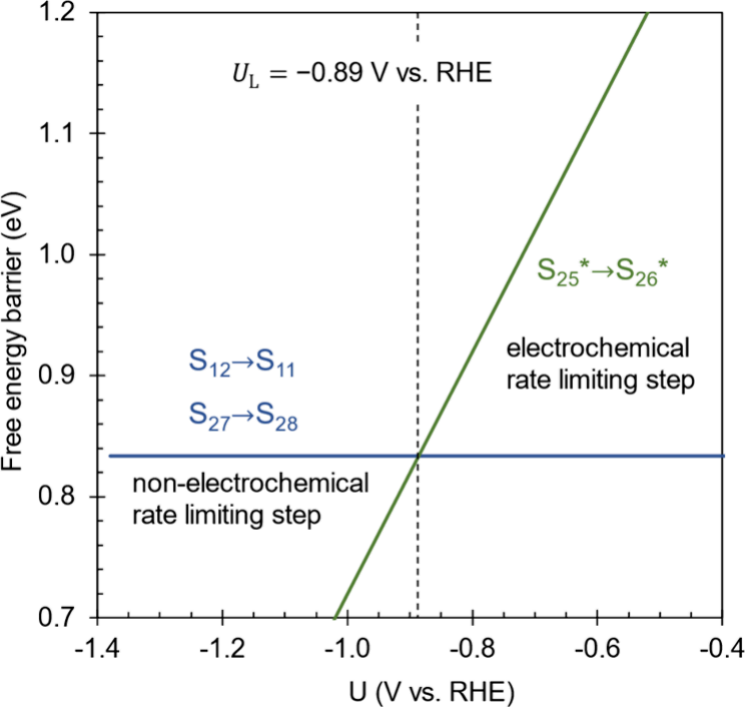
Total barriers for the possible rate limiting electrochemical (S_25^*^_ → S_26^*^_) and nonelectrochemical (S_12_ → S_11_ and S_27_ → S_28_) steps with respect to electrode potential. The limiting potential is indicated by the vertical dashed line and corresponds to the potential where the electrochemical step is on the cusp of becoming formally rate limiting.

The electrode potential affects both the reaction free energies and activation barriers of the two electron transfer steps in the catalytic cycle, S_25_ → S_25^*^_ and S_26^*^_ → S_26_. Because the activation barriers for these electron transfer steps are so low, the kinetically relevant effect of varying the electrode potential is due to changes in the free energies of subsequent intermediates and transition states. The only kinetically relevant total barrier that is dependent on the electrode potential is that of the proton transfer step S_25^*^_ → S_26^*^_. As depicted in Figure 8, a less cathodic electrode potential leads to an increase in the total barrier of this step, while the total barriers of the other formal rate limiting steps are unchanged. The intrinsic barrier for the proton transfer step is independent of the electrode potential since it does not involve any electron transfer. Instead, the influence of potential on the total barrier of this step is entirely due to the variation in free energy of the preceding intermediate S_25^*^_ with respect to the resting state S_24_. A more cathodic potential with respect to the RHE shifts the free energy of the reduced S_25^*^_ intermediate down closer to the free energy of the unreduced S_24_, leading to a decrease in the total barrier between S_24_ and the transition state of S_25^*^_ → S_26^*^_.

At sufficiently cathodic potentials, the total barrier for S_25^*^_ → S_26^*^_ is lower than the global barrier of 0.83 eV arising from the three other formal rate limiting steps S_12_ → S_11_, S_19_ → S_20_, and S_27_ → S_28_. As the potential becomes less cathodic, the total barrier of this step increases until it becomes equal to the global barrier at a potential of −0.89 V vs RHE. At this potential, S_25^*^_ → S_26^*^_ becomes formally rate limiting along with the three other steps. If the potential increases further, S_25^*^_ → S_26^*^_ becomes the sole formal rate limiting step and the global barrier now increases as the potential increases further. Since one electron is transferred between the global resting state S_24_ and the transition state of the rate limiting step S_25^*^_ → S_26^*^_, the transfer coefficient is 1.

The potential of −0.89 V vs RHE is thus not an “optimal” potential per se, but a limiting potential below which the TOF becomes independent of potential. A more cathodic potential will not increase the TOF since electron transfer is not kinetically involved in any of the rate limiting steps at these potentials.

### 7.3 Effect of Formaldehyde Concentration on the Free Energy Profile

We finally examine the effect of formaldehyde concentration on the kinetics of the isolated chain growth cycle. Technically, we are referring to the concentration of methanediol since this is the predominant form of formaldehyde in aqueous solution. The formaldehyde concentration can potentially affect the total barriers of all steps in the cycle up to dehydration (S_20_ → S_21_). This latter step is sufficiently irreversible that the total barriers of all subsequent steps are independent of the formaldehyde concentration.

**Figure 9:**
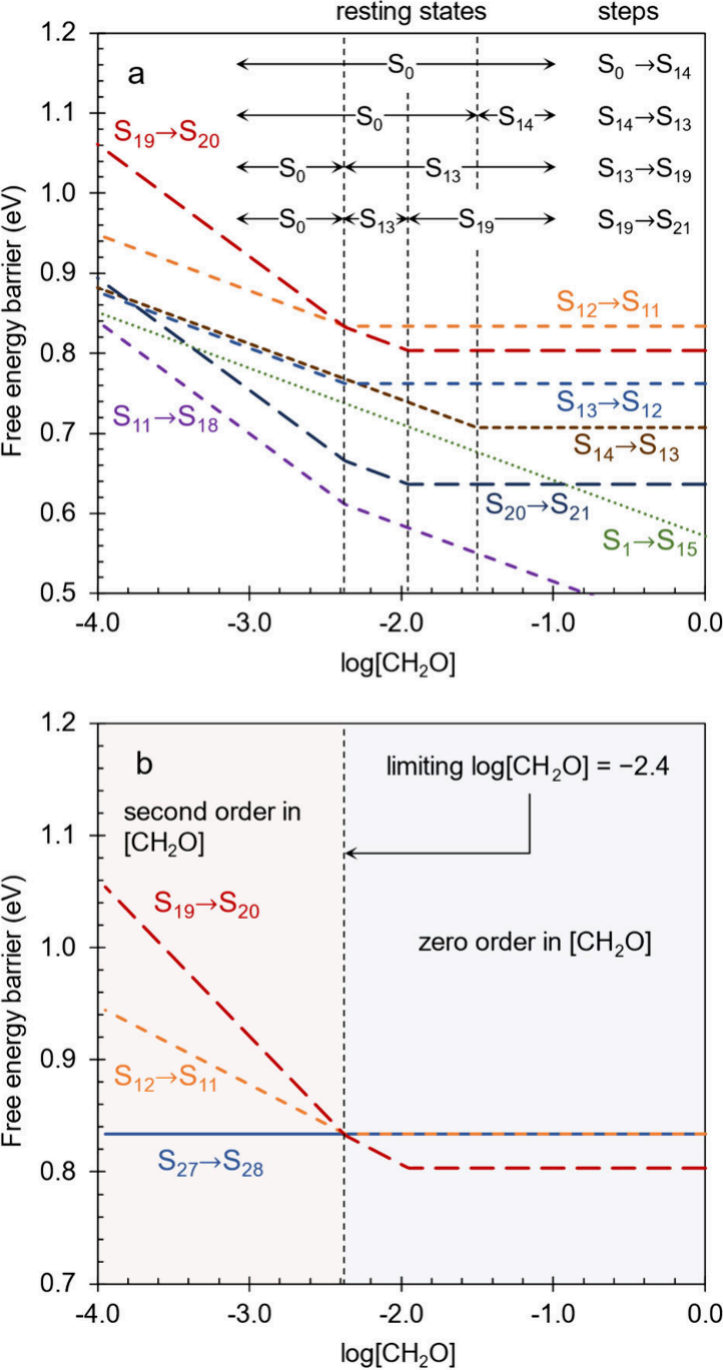
Total barriers for different steps in the chain growth cycle with respect to log[CH_2_O]. (a) The top panel indicates kinetic regimes defined by the kinetic dependence of these steps with respect to [CH_2_O]. Changes in the kinetic dependence of a step arise from changes in the resting state of that step as [CH_2_O] varies. (b) The bottom panel indicates kinetic regimes defined by the rate limiting steps, which are S_19_ → S_20_ at low [CH_2_O] (red) and S_12_ → S_11_/S_27_ → S_28_ at high [CH_2_O] (blue). The limiting log[CH_2_O] is defined as the lowest value at which the TOF is zero order in [CH_2_O]. In both panels, dotted lines indicate steps between S_0_ and S_14_, short dashed lines indicate the step S_14_ → S_13_, medium dashed lines indicate steps between S_13_ and S_19_, long dashed lines indicate steps between S_19_ and S_21_, and solid lines indicate steps after S_21_ (so that longer dashes correspond to later steps in the cycle). The steps are grouped according to the resting state they proceed from, as indicated at the top of the top panel.

The total barriers for all steps up to dehydration are depicted in Figure 9a with respect to formaldehyde concentration. The key point is that there are two ways in which the total barrier of a step depends on formaldehyde concentration–either through direct involvement of formaldehyde in the step or through quasi-equilibrium of a preceding step that involved formaldehyde addition. As [CH_2_O] increases, the latter dependence vanishes as the equilibria of all such preceding steps shifts toward the product. Thus, at high [CH_2_O], steps that directly involve formaldehyde addition (S_1_ → S_15_ and S_11_ → S_18_) exhibit a first order dependence on formaldehyde while all other steps exhibit zero order dependence.

To further explore the kinetic dependence of individual steps on formaldehyde concentration, the steps in Figure 9a have been divided into four groups depending on which resting state they proceed from S_1_ → S_14_, S_14_ → S_13_, steps between S_13_ and S_19_, and steps between S_19_ and S_21_ (dehydration). Additionally, the plot can be divided into four regimes based on the kinetic dependence of each group of steps with respect to [CH_2_O]. In the leftmost regime, corresponding to low [CH_2_O], all steps proceed from the resting state S_0_. The steps between S_1_ and S_11_ exhibit first order dependence on [CH_2_O] while the steps between S_11_ and S_21_ exhibit second order dependence. This dependence arises from the fact that one molecule of formaldehyde is transferred from the bulk electrolyte (in the formaldehyde addition step) to obtain the transition states for steps between S_1_ and S_11_ from the resting state S_0_, while two molecules of formaldehyde are transferred (one in formaldehyde addition and one in aldol addition) for the steps between S_11_ and S_21_.

At log[CH_2_O] of −2.4, intermediate S_13_ becomes lower in free energy than S_0_ so that the resting state for all steps between S_13_ and S_21_ shifts to S_13_. As a result, formation of the transition states for these steps from the new resting state S_13_ no longer requires transfer of formaldehyde from the bulk electrolyte in the formaldehyde addition step. Thus, the tautomerization steps between S_13_ and S_11_ become zero order in [CH_2_O], while the aldol addition and dehydration steps between S_11_ and S_21_ become first order. At log[CH_2_O] of −2.2, S_19_ becomes lower in free energy than S_13_ so that the resting state for the dehydration steps between S_19_ and S_21_ shifts from S_13_ to S_19_. Consequently, the dehydration steps no longer require transfer of formaldehyde from the bulk electrolyte to form their transition state from the resting state S_19_, eliminating their kinetic dependence on [CH_2_O]. Lastly at log[CH_2_O] of −1.5, S_14_ becomes lower in free energy than S_0_ so that the resting state for S_14_ → S_13_ shifts from S_0_ to S_14_ and this step also becomes zero order in [CH_2_O].

Figure 9b examines how the rate limiting step changes with respect to [CH_2_O]. At low [CH_2_O], the deprotonation step involved in dehydration, S_19_ → S_20_, is rate limiting but switches to S_12_ → S_11_ and S_27_ → S_28_ (the deprotonation steps involved in tautomerizations) at high [CH_2_O]. In the low [CH_2_O] regime, the rate limiting step (S_19_ → S_20_) requires the transfer of two molecules of formaldehyde from the bulk electrolyte to form its transition state from the resting state S_0_, leading to a second order overall dependence on [CH_2_O]. At the formaldehyde concentration increases, the total barrier for this step rapidly decreases until the tautomerization steps (S_12_ → S_11_ and S_27_ → S_28_) become rate limiting at the limiting log[CH_2_O] of −2.4. Since both of these steps proceed from their respective resting states (S_13_ and S_27_) without involving formaldehyde, they have no kinetic dependence on [CH_2_O] so that the overall kinetics are zero order in formaldehyde with a constant global barrier of 0.83 eV. This defines the limiting value of log[CH_2_O] of −2.4, which represents the lowest formaldehyde concentration before the TOF begins to decrease, analogous to how the limiting potential represents the highest potential before the TOF begins to decrease. Coincidentally, the rate limiting step switches from S_19_ → S_20_ to S_12_ → S_11_ at the exact same value of log[CH_2_O] = −2.4 that the resting state switches from S_0_ to S_13_. This is independent of pH since both steps are deprotonation and proceed from unprotonated resting states.

## 8 Coupling of the Chain Growth and CO_2_ Reduction Cycles

**Figure 10:**
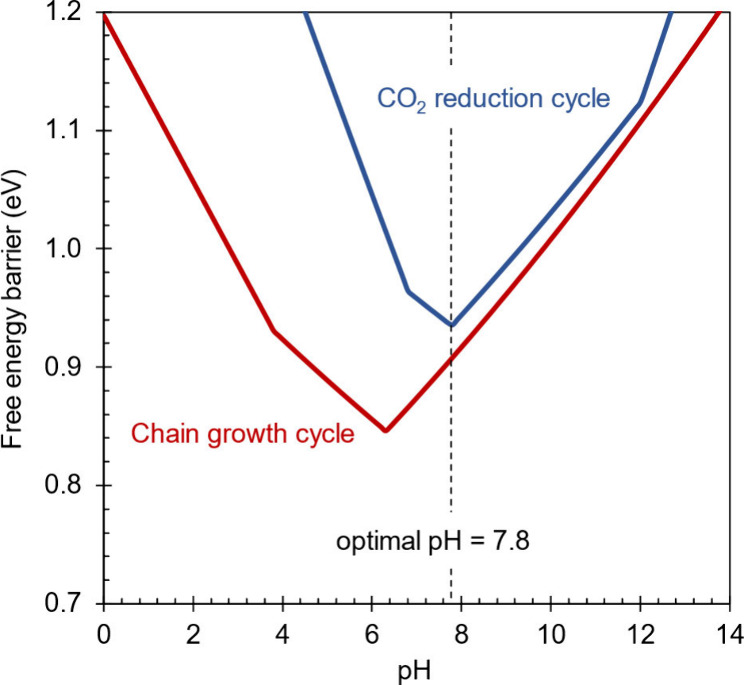
Limiting global barriers for the CO_2_ reduction and chain growth cycles with respect to electrolyte pH. The limiting global barriers correspond to electrode potentials more cathodic than the limiting potential and formaldehyde concentrations below (CO_2_ reduction) or above (chain growth) the limiting concentration.

We now discuss the kinetic coupling between the CO_2_ reduction cycle and the chain growth cycle. These two cycles are coupled by the formaldehyde concentration since the kinetics of both potentially depend on it. When the formaldehyde concentration is at steady state, the TOF of the CO_2_ reduction cycle will be twice the TOF of the chain growth cycle since the former produces one molecule of formaldehyde while the latter consumes two molecules. Also, when the two cycles are coupled they must operate at the same pH. The limiting global barrier of each cycle, defined as the global barrier when the respective cycle is not kinetically limited by either potential or formaldehyde concentration, is plotted in Figure 10 with respect to pH. At all values of pH, the limiting global barrier of the CO_2_ reduction cycle is higher than the limiting global barrier of the chain growth cycle. Therefore, the optimal pH for the coupled cycle is determined by the optimal pH of the CO_2_ reduction cycle, which was determined to be 7.8 in our previous study.^8^

At this pH, the CO_2_ reduction cycle is zero order in formaldehyde when log[CH_2_O] is lower than −2.5. Above this concentration, the global resting state shifts from S_1_ to S_13_ so that the cycle is inhibited to first order by formaldehyde. The kinetic dependence of the chain growth cycle on formaldehyde concentration at this same pH is depicted in Figure S4 in the Supporting Information, where it can be seen that the behavior is qualitatively similar to the behavior at a pH of 6.7 (the optimal pH of the isolated chain growth cycle). One can see from this figure that the chain growth cycle is second order in formaldehyde when log[CH_2_O] is less than −2.5 and the rate limiting step is the deprotonation step S_19_ → S_20_ proceeding from the resting state S_1_. Above this concentration, the rate limiting step shifts to the deprotonation step S_12_ → S_11_ proceeding from resting state S_13_ and is thus kinetically independent of formaldehyde concentration. As mentioned in Section 7.3, it is purely a coincidence that the rate limiting step and the resting state switch at exactly the same value of log[CH_2_O].

When log[CH_2_O] is equal to −2.5 neither cycle is limited by [CH_2_O] since S_1_ and S_13_ have the same free energy. At these conditions, the chain growth cycle has a TOF that is about 3.5 times higher (1.2 s^–1^) than the CO_2_ reduction cycle (0.34 s^–1^). Steady state is reached when this ratio is 0.5 and occurs at log[CH_2_O] of −3.1. At this formaldehyde concentration, the CO_2_ reduction cycle is still operating at its limiting TOF of 0.34 s^–1^, while the chain growth cycle has slowed to a TOF of 0.17 s^–1^. The free energy diagrams for both the CO_2_ reduction cycle and the chain growth cycle at these conditions are depicted in Figure S5 in the Supporting Information.

## 9 Conclusions

In summary, we have computationally demonstrated that the electro-organocatalyst designed in our previous work for carrying out electrochemical CO_2_ reduction to formaldehyde should also be capable of electrochemically coupling the formaldehyde into long chain aldehydes. The key feature of the catalyst is a vicinal enediamine (>N–C=C–N<) catalytic motif that activates formaldehyde to function as a nucleophile in aldol addition with a second aldehyde while subsequently allowing for efficient electron transfer from a chemically inert cathode along with facile tautomerization.

The mechanism begins with addition of formaldehyde to the vicinal enediamine. The electron-rich π system that results from having two nitrogen atoms adjacent to the C=C bond reverses the polarity of the carbon atom in formaldehyde, allowing it to act as a nucleophile in the subsequent aldol addition condensation step. This is then followed by a sequence of two proton coupled electron transfer (PCET) steps where the electron transfers occur by an outer sphere mechanism from a chemically inert cathode. The PCET mechanism is analogous to the second PCET step in the CO_2_ reduction cycle, being facilitated by the ability of the >N^+^=C–CH=O π system to avoid unfavorable placement of formal charge on either of the carbon atoms; the two electrons instead formally transfer to the nitrogen and oxygen atoms. Tautomerization and dehydration steps are also facilitated by the ability of the electron-rich nitrogen atoms to accommodate formal positive charge. The cycle ends with elimination of an aldehyde that occurs analogously to the initial formaldehyde addition step.

The catalytic cycle is found to give the highest turnover frequency of 9.3 s^–1^ at a pH of 6.7 and a limiting electrode potential of −0.89 V vs RHE. The optimal pH is determined by a balance between rate limiting protonation and deprotonation steps while the limiting potential is determined by a balance between rate limiting electrochemical and nonelectrochemical steps. The chain growth cycle, which consumes formaldehyde, is found to be kinetically faster than the CO_2_ reduction cycle that produces the formaldehyde. Therefore, the optimal pH for coupling the two cycles is 7.8 which is the optimal pH for operating the CO_2_ reduction cycle. When both cycles are coupled under steady state conditions, the turnover frequency of the chain growth cycle is predicted to be 0.17 s^–1^.

## Supporting Information Available

Technical details of the DFT calculations, description of the hybrid solvation method, method for finding electron transfer transition states, corrections to DFT energies, free energy relation for extrapolating proton transfer barriers, details of benchmarking calculations in NWChem for hybrid DFT functionals, free energy contributions of intermediates and transition states, the pH dependence of the total barriers of all other steps in the catalytic cycle, the kinetic dependence of the chain growth cycle on formaldehyde concentration at pH of 7.8, the free energy diagrams for both the CO_2_ reduction cycle and the chain growth cycle under optimal coupling conditions, structures and hydrogen bonding free energies of intermediates and transition states, free energy contributions for intermediates and transition states (PDF)
