# Supplementary material for: Computational Design of an Electro-Organocatalyst for Conversion of CO2 into Long Chain Aldehydes
Source: J Phys Chem A. 2024 Jul 4;128(28):5445–58. doi: 10.1021/acs.jpca.4c00780 (PMC11264266; doi:10.1021/acs.jpca.4c00780)
Supplement: Supplementary file 1 — jp4c00780_si_001.pdf [file jp4c00780_si_001.pdf]

Computational Design of an Electro-Organocatalyst for Conversion of CO_2 into Long Chain Aldehydes

Supporting Information

Foroogh Khezeli[†] and Craig Plaisance[†,*]

[†]Cain Department of Chemical Engineering, Louisiana State University, Baton Rouge, Louisiana 70803, United States

1. Technical details of the DFT calculations (reproduced from ref [1])

Density functional theory calculations were performed using the Vienna Ab-initio Simulation Package (VASP)[2] combined with the VASPsol extension for including an implicit electrolyte in the simulation[3,4]. The exchange-correlation energy was computed at the generalized gradient approximation level using the Bayesian error estimation functional with van der Waals correlation (BEEF-vdW)[5]. The wave functions were constructed using plane waves with an energy up to 400 eV, while the projector augmented wave (PAW) method[6,7] was used to represent the oscillations in the core region. The Kohn-Sham orbital populations were determined using an error function distribution having a width of 0.2 eV and the energy was extrapolated to zero temperature. All species were placed in a 20×20×20 Å unit cell and only the Γ-point was used to sample the Brillouin zone.

All minimum energy and transition state structures were optimized using a conjugate gradient method until the forces on all atoms were below 0.05 eV/Å. The dimer method[8] was used to identify the negative curvature mode in transition states and reverse the force in this direction during the optimization. A distance of 0.05 Å was used to separate the central image from the dimer image. In most cases, the nudged elastic band method [9,10] was used to obtain an initial guess for the transition state structure and negative curvature mode prior to running dimer calculations. These calculations typically employed eight images along the reaction pathway and were converged to a maximum atomic force of 0.3 eV/Å. A special method was used to optimize transition states of electron transfer reactions, which is discussed in **Section 0**.

Vibrational frequencies of all structures were determined from the atomic Hessian using the harmonic approximation. The atomic Hessian was constructed using the finite difference technique by displacing each atom by 0.015 Å in all six Cartesian directions. All transition states were verified to be saddle points on the potential energy surface by the presence of one imaginary frequency mode. The lowest six (five) frequencies were discarded for polyatomic (diatomic/linear) molecules as these correspond to rotation and translation. For transition states, the six lowest frequencies excluding the imaginary frequency reaction mode were discarded.

2. Description of the hybrid solvation method (reproduced from ref [1])

The hybrid solvation method combines an implicit continuum description of the bulk electrolyte along with the inclusion of a certain number of explicit water molecules to more accurately account for strong hydrogen bonds formed by the intermediates and transition states. The implicit part of the electrolyte is modeled by a linear dielectric and ionic response using the VASPsol extension to VASP developed by the Hennig group[3,4]. The dielectric response is represented by a dielectric constant of 78.4 corresponding to bulk water at 25°C, while the ionic response is represented by a Debye length of 3 Å corresponding to a 1:1 electrolyte at a concentration 1 mol/L.

The free energy of a species in the electrolyte that is hydrogen bonded to n_{W} explicit water molecules is given by,

$$G^{\circ} = E_{0,\mathrm{aq}} + E_{\mathrm{ZPVE}} + G^{\circ}_{\mathrm{trans,aq}} + G_{\mathrm{rot}} + G_{\mathrm{vib}} - n_{\mathrm{W}}\mu_{\mathrm{W}} \quad \text{(S1)}$$

where $E_{0,\mathrm{aq}}$ is the electronic energy extrapolated to zero temperature plus the implicit solvation free energy reported by VASPsol. The translational free energy $G^{\circ}_{\mathrm{trans,aq}}$ is computed at the standard state concentration of 1 mol/L, while the rotational free energy G_{rot} is computed using the rigid rotor approximation. The vibrational free energy G_{vib} along with the zero-point vibrational energy E_{ZPVE} are computed within the harmonic approximation using the frequencies computed by VASP. One difficulty encountered with including explicit water molecules in a calculation is that there are very loose vibrational modes associated with translational and rotational motion of these waters. Consequently, the harmonic approximation is not appropriate for computing the contribution of these motions to the partition function. To avoid this difficulty, the translational, rotational, and vibrational contributions are computed for each species in the absence of explicit water molecules, using only implicit solvation. The vibrational contributions to the free energy from motion of the explicit water molecules is accounted for empirically in the explicit water chemical potential μ_{W}.

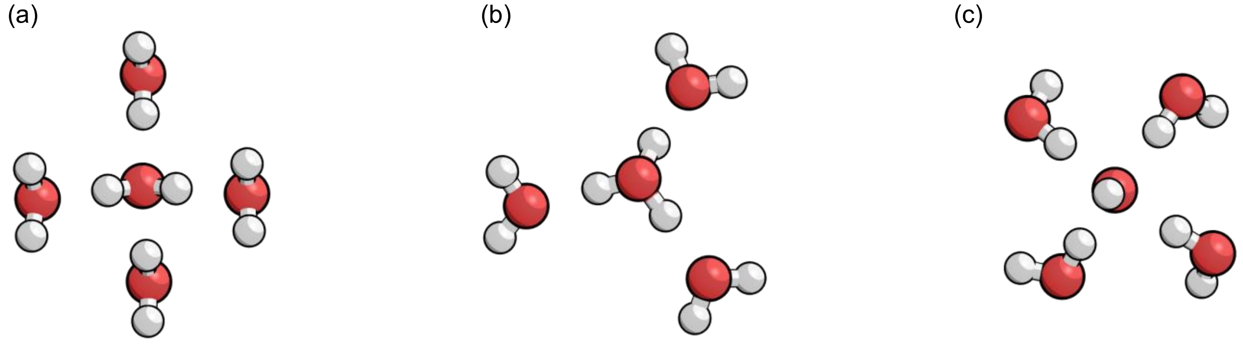

Figure S1. Structures of (a) H_2O hydrogen bonded to four explicit waters, (b) H_3O^+ hydrogen bonded to three explicit waters, and (c) OH^- hydrogen bonded to four explicit waters.

To compute μ_{W}, an empirical correction $G_{\mathrm{W,corr}}$ is added to the electronic energy $E_{\mathrm{W,aq}}$ of a water molecule 'solvated' in the implicit electrolyte,

(S2)

$$\mu_{\mathrm{W}} = E_{\mathrm{W,aq}} + G_{\mathrm{W,corr}}$$

The empirical correction is obtained by requiring that the free energy of the 'solvated' molecule of water is the same as for a molecule of water in the saturated vapor phase, ensuring thermodynamic consistency between the description of water in both phases. The 'solvated' molecule of water is hydrogen bonded to four explicit water molecules in the configuration shown in **Figure S1**a. Its free energy is computed by eq (S1) with an additional term accounting for the experimental concentration of liquid water $C_{\mathrm{W}} = 55.6$ mol/L. This leads to the condition,

(S3)

$$G_{\mathrm{W,corr}} = \frac{1}{4}\left[G^{\circ}_{\mathrm{H_2O,aq}} + k_{\mathrm{B}}T\ln\frac{C_{\mathrm{W}}}{C^{\circ}} - \mu_{\mathrm{H_2O}}\right] - E_{\mathrm{W,aq}}$$

where the chemical potential of water corresponds to an ideal gas at the experimental saturation pressure P_{sat} (0.474 bar at 80°C),

(S4)

$$\mu_{\mathrm{H_2O}} = G^{\circ}_{\mathrm{H_2O,g}} + k_{\mathrm{B}}T\ln\frac{P_{\mathrm{sat}}}{P^{\circ}}$$

The ideal gas free energy of water and other species is calculated by,

(S5)

$$G^{\circ} = E_{0,\mathrm{g}} + E_{\mathrm{ZPVE}} + G^{\circ}_{\mathrm{trans,g}} + G_{\mathrm{rot}} + G_{\mathrm{vib}}$$

where the electronic energy $E_{0,\mathrm{g}}$ is computed for the molecule in vacuum (without implicit solvation) and the translational free energy is computed at the standard pressure P° of 1 bar. Using the calculated free energies in **Table S1**, the empirical correction is computed to be 0.126 eV per explicit water molecule.

Table S1. Free energy contributions (eV) for species used in the solvation model.

	$\boldsymbol{E_0}$	$\boldsymbol{E_{\mathrm{ZPVE}}}$	$\boldsymbol{G_{\mathrm{trv}}}$
H_2 (g)	-7.15	0.27	-0.39
H_2O (g)	-12.81	0.57	-0.59
H_2O (aq)	-13.14	0.57	-0.49
$H_2O \cdot 4W$ (aq)	-66.11	0.57	-0.49
$H_3O^+ \cdot 3W$ (aq)	-52.13	0.91	-0.50
$OH^- \cdot 4W$ (aq)	-65.79	0.24	-0.44

It is also necessary to define the chemical potential of the proton within the solvation method. This is defined by considering the process by which water is protonated to give hydronium,

$$\mathrm{H_2O + H^+ \rightleftharpoons H_3O^+}$$

By definition, this process should be equilibrated at zero pH when the hydronium activity is unity. This leads to the condition,

(S6)

$$\mu_{\mathrm{H^+}}^{\circ} = G_{\mathrm{H_3O^+,aq}}^{\circ} - \mu_{\mathrm{H_2O}}$$

For the proton chemical potential at zero pH. The value computed from the energies in **Table S1** is $\mu_{\mathrm{H^+}}^{\circ} = 0.93$ eV, which is based on a hydronium ion hydrogen bonded to three explicit water molecules in an Eigen configuration as depicted in **Figure S1**b. We can also compute the equilibrium constant for autoionization of water,

$$2\,\mathrm{H_2O} \rightleftharpoons \mathrm{H_3O^+} + \mathrm{OH^-}$$

according to,

$$\mathrm{p}K_{\mathrm{W}} = -\frac{1}{k_{\mathrm{B}}T\ln 10}\left[G_{\mathrm{H_3O^+,aq}}^{\circ} + G_{\mathrm{OH^-,aq}}^{\circ} - 2\,\mu_{\mathrm{H_2O}}\right] \qquad \text{(S7)}$$

The value calculated from the energies in **Table S1** is 11.9, in excellent agreement with the experimental value of 12.5 at 80°C. This was computed based on a hydroxide ion hydrogen bonded to four explicit water molecules as depicted in **Figure S1**c.

Finally, the electron chemical potential in the standard hydrogen electrode can be computed as the value at which the process,

$$\frac{1}{2}\mathrm{H_{2\,(g)}} \rightleftharpoons \mathrm{H^+} + \mathrm{e^-}$$

is equilibrated at zero pH and an H_2 partial pressure of 1 bar. This leads to the condition,

$$\mu_{\mathrm{e^-,SHE}} = \frac{1}{2}G_{\mathrm{H_2,g}}^{\circ} - \mu_{\mathrm{H^+}}^{\circ} \qquad \text{(S8)}$$

Using the energies in **Table S1**, the electron chemical potential in the standard hydrogen electrode is computed to be −4.56 eV with respect to vacuum which is within the range of experimentally measured values.

3. Method for finding electron transfer transition states (reproduced from ref [1])

The method we implemented in VASP for locating an intersystem crossing between the potential energy surfaces of two electronic states is adapted from the method of Bearpark et al. for locating a conical intersection[11]. The only difference is that we neglect Hamiltonian coupling between the two states, as this is outside the context of the Kohn-Sham formalism. This method is implemented in VASP by running calculations of two images (labeled 1 and 2) in parallel that have the same geometric structure but different numbers of electrons. Because the two images have different electronic structures, the forces on the atoms in each one will be different. We define the $3N$ dimensional (N is the number of atoms in the system) reaction coordinate $\mathbf{g}$ as the normalized difference in these force vectors $\mathbf{F}_1$ and $\mathbf{F}_2$,

(S9)

$$\mathbf{g} = \frac{\mathbf{F}_1 - \mathbf{F}_2}{||\mathbf{F}_1 - \mathbf{F}_2||}$$

We then project out the force along this direction from either of the two images (the result is independent of which image we use) and add a force back along this direction that is proportional to the difference between the energies of the two electronic states,

(S10)

$$\mathbf{F} = \mathbf{F}_2 - (\mathbf{F}_2 \cdot \mathbf{g})\,\mathbf{g} + \alpha\left[(E_1 - E_2) - \left(n_{\mathrm{e}^-,1} - n_{\mathrm{e}^-,2}\right)\mu_{\mathrm{e}^-}\right]\mathbf{g}$$

Here, E_1 and E_2 are the electronic energies while $n_{\mathrm{e}^-,1}$ and $n_{\mathrm{e}^-,2}$ are the numbers of electrons in the two images. The electron chemical potential μ_{e^-} must also be specified and is related to the electrode potential U_{SHE} by,

(S11)

$$\mu_{\mathrm{e}^-} = \mu_{\mathrm{e}^-,\mathrm{SHE}} - U_{\mathrm{SHE}}$$

where $\mu_{\mathrm{e}^-,\mathrm{SHE}}$ is given by eq (S8). The resulting force vector $\mathbf{F}$ is used with a conjugate gradient algorithm to locate the transition state. The parameter α is used to adjust the weight of the force along the reaction coordinate and was always set to a value of 10 Å^{-1}.

4. Corrections to DFT energies (reproduced from ref [1])

To correct for intrinsic errors in the DFT energies of the reactant and product molecules, we employ empirical corrections for different functional groups that appear in the various intermediates in the catalytic cycle. Specifically, corrections are applied to the CO_2 molecule, the carboxyl/carboxylate group and its enediol tautomer, the carbonyl group and its enol tautomer, and the gem diol group. These corrections are based on the calculated and experimental enthalpies of gas phase reactions that combine methanol and water to form CO_2, formic acid, formaldehyde, and methanediol,

$$\mathrm{CH_3OH + H_2O \rightarrow CO_2 + 3\ H_2}$$

$$\mathrm{CH_3OH + H_2O \rightarrow HCO_2H + 2\ H_2}$$

$$\mathrm{CH_3OH \rightarrow H_2CO + H_2}$$

$$\mathrm{CH_3OH + H_2O \rightarrow CH_2(OH)_2 + H_2}$$

The DFT enthalpies of the reactant and product molecules are given in **Table S2** and are computed by,

$$H = E_0 + E_{\mathrm{ZPVE}} + H_{\mathrm{vib}} + \left(\frac{m}{2} + 1\right) k_{\mathrm{B}} T \qquad \text{(S12)}$$

where m is the number of translational and rotational degrees of freedom ($m = 6$ for polyatomic molecules and $m = 5$ for diatomic and linear molecules). The vibrational enthalpy H_{vib} and zero-point vibration energy E_{ZPVE} are computed within the harmonic approximation. The enthalpy contributions for the molecules are given in **Table S1** while the calculated and experimental reaction enthalpies are reported in **Table S3**. The corrections to DFT energies of each functional group are reported in **Figure S2**.

Table S2. Enthalpy contributions (eV) from DFT for molecules used in reactions to determine DFT corrections.

	$\boldsymbol{E_0}$	$\boldsymbol{E_{\mathrm{ZPVE}}}$	$\boldsymbol{H_{\mathrm{vib}}}$
H_2O	-12.81	0.57	0.00
H_2	-7.15	0.27	0.00
CH_3OH	-27.74	1.38	0.02
$CH_2(OH)_2$	-32.85	1.53	0.04
H_2CO	-19.55	0.71	0.00
HCO_2H	-25.52	0.90	0.02
CO_2	-18.44	0.31	0.01

Table S3. DFT and experimental reaction enthalpies (eV) used to determine DFT corrections.

	$\boldsymbol{\Delta H_{\mathrm{r,DFT}}}$	$\boldsymbol{\Delta H_{\mathrm{r,expt}}}$	$\boldsymbol{E_{\mathrm{corr}}}$
$\mathbf{CH_3OH + H_2O \rightarrow CO_2 + 3\ H_2}$	-0.01	0.51	0.52
$\mathbf{CH_3OH + H_2O \rightarrow HCO_2H + 2\ H_2}$	0.29	0.67	0.37
$\mathbf{CH_3OH \rightarrow H_2CO + H_2}$	0.72	0.95	0.23
$\mathbf{CH_3OH + H_2O \rightarrow CH_2(OH)_2 + H_2}$	0.41	0.51	0.11

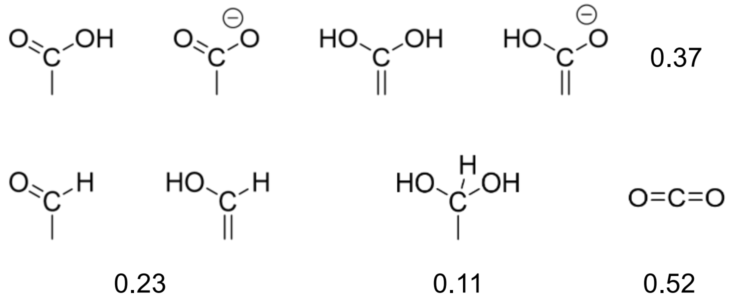

Figure S2. Corrections to DFT energies (eV) for different functional groups.

5. Free energy relation for extrapolating proton transfer barriers (partially reproduced from ref [1])

The intrinsic activation barrier for a step in which intermediate S_i is protonated by a PTM to give S_j is given by,

(S13)

$$\Delta^{\mathrm{a,i}}G^{\circ}_{i\to j} = \frac{\left(\Delta^{\mathrm{r,i}}G^{\circ}_{i\to j} - \beta_{\mathrm{HA}} + \beta_{\mathrm{A^-}} + \lambda_{i\to j}\right)^2}{4\lambda_{i\to j}} + \beta_{\mathrm{HA}}$$

The intrinsic reaction free energy used in this expression is given in terms of the product acidity $\mathrm{p}K_{\mathrm{a},j}$ and the PTM acidity $\mathrm{p}K_{\mathrm{a}}$,

(S14)

$$\Delta^{\mathrm{r,i}}G^{\circ}_{i\to j} = k_{\mathrm{B}}T\ln 10 \times \left(\mathrm{p}K_{\mathrm{a}} - \mathrm{p}K_{\mathrm{a},j}\right)$$

The intrinsic reaction free energies and barriers calculated using formic acid as the PTM are reported in **Table S4** along with the corresponding values of λ and $\mathrm{p}K_{\mathrm{a},j}$.

Table S4. Intrinsic reaction free energies, intrinsic activation barriers, reorganization energies, and product pK_a values for protonation steps. Calculated for a formic acid PTM with pK_a of 3.3.

	$\Delta^{\mathrm{r,i}}G^{\circ}$	$\Delta^{\mathrm{a,i}}G^{\circ}$	λ	$\mathrm{p}K_{\mathrm{a},j}$
$S_{13}\to S_{12}$	-0.17	0.63	0.97	5.71
$S_{21}\to S_{22}$	-0.22	0.59	0.88	6.39
$S^*_{25}\to S^*_{26}$	-0.52	0.41	0.40	12.60
$S_{26}\to S_{27}$	-0.35	0.56	0.98	8.31
$S_{28}\to S_{29}$	-0.13	0.73	1.30	5.22

The intrinsic activation barrier for a step in which intermediate S_i is deprotonated by a PTM to give S_j is given by,

(S15)

$$\Delta^{\mathrm{a,i}}G^{\circ}_{i\to j} = \frac{\left(\Delta^{\mathrm{r,i}}G^{\circ}_{i\to j} - \beta_{\mathrm{A^-}} + \beta_{\mathrm{HA}} + \lambda_{i\to j}\right)^2}{4\lambda_{i\to j}} + \beta_{\mathrm{A^-}}$$

The intrinsic reaction free energy used in this expression is given in terms of the reactant acidity $\mathrm{p}K_{\mathrm{a},j}$ and the PTM acidity $\mathrm{p}K_{\mathrm{a}}$,

(S16)

$$\Delta^{\mathrm{r,i}}G^{\circ}_{i\to j} = k_{\mathrm{B}}T\ln 10 \times \left(\mathrm{p}K_{\mathrm{a},i} - \mathrm{p}K_{\mathrm{a}}\right)$$

The intrinsic reaction free energies and barriers calculated using formic acid as the PTM are reported in **Table S5** along with the corresponding values of λ and $\mathrm{p}K_{\mathrm{a},i}$.

Table S5. Intrinsic reaction free energies, intrinsic activation barriers, reorganization energies, and reactant pK_a values for deprotonation steps. Calculated for a formate PTM with pK_a of 3.3.

	$\Delta^{r,i}G^\circ$	$\Delta^{a,i}G^\circ$	λ	p$K_{a,i}$
S_{14}→S_{13}	0.18	0.82	1.02	5.83
S_{12}→S_{11}	0.35	0.90	0.94	8.23
S_{19}→S_{20}	0.47	0.95	0.84	9.97
S_{22}→S_{24}	-0.51	0.69	1.64	-4.02
S_{27}→S_{28}	0.19	0.95	1.51	6.01

6. Details of benchmarking calculations in NWChem for hybrid DFT functionals

In order to benchmark the quality of the proton transfer barriers reported in the manuscript, we have performed additional calculations in the NWChem code[12] using the PBE0[13] and B3LYP[14] hybrid DFT functionals. This was done for two reaction steps: $S_3 \rightarrow S_4$ in the CO_2 reduction cycle and $S_{19} \rightarrow S_{20}$ in the chain growth cycle. Both of these are rate limiting steps in their respective cycles and thus provide a reasonable benchmark for the overall accuracy of the calculated turnover frequencies.

Specifically, calculations were performed on the intermediates S_3, S_4, S_{19}, and S_{20} and the transition states $S_3 \rightarrow S_4$ and $S_{19} \rightarrow S_{20}$. Additionally, calculations were performed on the model proton transfer mediators (PTMs) formic acid and formate. The structures of all intermediates were optimized using the default algorithm and with the same DFT functional used to calculate the energy. Transition state structures were optimized using the saddle point optimization task in NWChem. Initial guesses of all structures were taken from the original calculations performed in VASP using the BEEF-vdW[5] functional. All calculations employed the 6-31g** basis set.

Solvation was accounted for using the COSMO solvation model[15,16] with a dielectric constant of 78.0 and the default parameters for atomic radii. The optimal number of explicit water molecules was also included in each structure, as determined in our previous work[1] for $S_3 \rightarrow S_4$ and in this work for $S_{19} \rightarrow S_{20}$. To utilize the appropriate reference for the explicit waters, we used the same approach employed in our VASP calculations outlined in **Section S2** by determining an empirical hydrogen bonding correction for explicit water. This entailed performing additional calculations on a single implicitly solvated water molecule, the explicitly solvated water depicted in Figure S1(a), and a single water molecule in the gas phase. The resulting values for the empirical correction $G_{\mathrm{W,corr}}$ are given in **Table S6** for the different functionals used in NWChem. Additionally, this value was computed for the PBE functional[17] in VASP with the VASPsol[3,4] solvation model.

Comparison with VASP results performed using the BEEF-vdW functional is somewhat complicated since the two codes utilize different solvation models (VASPsol in VASP and COSMO in NWChem). In order to make a reliable comparison, we examine the change in the reorganization energy λ described in **Section S5** instead of the change in the activation barrier. This allows us to isolate the effect of the functional on the activation barrier from its effect on the reaction energy.

A second complication arises because the BEEF-vdW functional is not available in NWChem. To circumvent this, we use the PBE functional as a basis for comparison since it is implemented in both NWChem and VASP. The correction to the reorganization energy is computed as,

(S17)

$$\Delta\lambda = \lambda(\text{hybrid,NWChem}) - \lambda(\text{PBE,NWChem}) + \lambda(\text{PBE,VASP}) - \lambda(\text{BEEF-vdW,VASP})$$

where λ for the reaction is computed using a hybrid functional (PBE0 or B3LYP) and the PBE functional in NWChem and using the PBE and BEEF-vdW functionals in VASP. The intrinsic activation barriers and intrinsic reaction free energies used to determine the λ values in eq (S17) do not include zero-point vibrational energy or thermal free energy corrections since these are unlikely to be affected significantly by the choice of functional. Once $\Delta\lambda_{3\rightarrow4}$ is determined, it is added to the original value of $\lambda_{3\rightarrow4}$ (1.51 eV[1]) to obtain a corrected value for $\lambda_{3\rightarrow4}$. The corrected $\lambda_{3\rightarrow4}$ is then used to compute a corrected activation barrier for the protonation step $S_3 \rightarrow S_4$ according to eqs (S13) and (S14), using values of $\Delta^{\mathrm{a,i}}G^{\circ}_{3\rightarrow4} = 0.52$

eV and $\Delta^{\mathrm{r,i}}G^{\circ}_{3\to4} = 0.27$ eV as reported in ref [1]. The corrected activation barrier for the deprotonation step $S_{19}\to S_{20}$ can be computed in a similar way using eqs (S15) and (S16) and the parameters for this step in **Table S5**.

The results are presented in **Table S6**. We find that the barrier for $S_3\to S_4$ decreases by −0.01 eV when using the PBE0 functional and increases by 0.08 eV when using the B3LYP functional. The barrier for $S_{19}\to S_{20}$ increases by 0.07 eV when using the PBE0 functional and decreases by −0.02 eV when using the B3LYP functional. The increase is less than expected because the BEEF-vdW functional performs better for calculating proton transfer barriers than the more widely used PBE functional. This is in line with findings that functionals including nonlocal van der Waals interactions perform better for calculating proton transfer barrier than standard GGA functionals.[18] Specifically, we find that the BEEF-vdW barrier for $S_3\to S_4$ is higher than the PBE barrier by 0.14 eV, while the BEEF-vdW barrier for $S_{19}\to S_{20}$ is higher than the PBE barrier by 0.08 eV. This means that the PBE0 and B3LYP barriers for $S_3\to S_4$ are higher than the PBE barrier by 0.13 and 0.22 eV, while the PBE0 and B3LYP barriers for $S_{19}\to S_{20}$ are higher than the PBE barrier by 0.15 and 0.06 eV. The differences between PBE and the hybrid functionals are in line with differences reported for other proton transfer barriers when comparing GGA and hybrid functionals.[18]

Table S6. Intrinsic activation barriers ($\Delta^{\mathrm{a,i}}G^{\circ}_{i\to j}$), intrinsic reaction free energies ($\Delta^{\mathrm{r,i}}G^{\circ}_{i\to j}$), reorganization energies ($\lambda_{i\to j}$), corrections to reorganization energies ($\Delta\lambda_{i\to j}$), and corrections to activation barriers for $S_3\to S_4$ and $S_{19}\to S_{20}$. The empirical hydrogen bonding corrections for explicit water ($G_{\mathrm{W,corr}}$) are also reported. All values are in eV.

	**BEEF-vdW (VASP)**	**PBE (VASP)**	**PBE (NWChem)**	**PBE0 (NWChem)**	**B3LYP (NWChem)**
$\mathbf{S_3\to S_4}$					
$\boldsymbol{\Delta^{\mathrm{a,i}}G^{\circ}_{3\to4}}$	0.43	0.25	0.28	0.28	0.38
$\boldsymbol{\Delta^{\mathrm{r,i}}G^{\circ}_{3\to4}}$	-0.03	-0.11	0.14	-0.13	-0.12
$\boldsymbol{\lambda_{3\to4}}$	1.78	1.19	0.82	1.38	1.74
$\boldsymbol{\Delta\lambda_{3\to4}}$				-0.03	0.33
**correction**				**-0.01**	**0.08**
$\mathbf{S_{19}\to S_{20}}$					
$\boldsymbol{\Delta^{\mathrm{a,i}}G^{\circ}_{19\to20}}$	0.38	0.23	0.33	0.52	0.33
$\boldsymbol{\Delta^{\mathrm{r,i}}G^{\circ}_{19\to20}}$	0.12	-0.01	0.02	0.09	-0.11
$\boldsymbol{\lambda_{19\to20}}$	1.28	0.95	1.28	1.91	1.55
$\boldsymbol{\Delta\lambda_{19\to20}}$				0.30	-0.06
**correction**				**0.07**	**-0.02**
$\boldsymbol{G_{\mathrm{W,corr}}}$	0.13	0.18	0.32	0.27	0.27

7. Free energy contributions of intermediates and transition states

Table S7. Free energy contributions (eV) for reactant and product molecules. Standard conditions of 1 bar (CO_2) and 1 mol/L (aqueous species) were used. The n_W column indicates the optimal number of explicit hydrogen bonded waters (giving the lowest free energy), while $\Delta G_\mathrm{W} = E_{0,\mathrm{aq},n_\mathrm{W}} - E_{0,\mathrm{aq}} - n_\mathrm{W}\mu_\mathrm{W}$ contains the interaction free energy between the molecule and the explicit waters.

	E_0	E_{ZPVE}	G_{trv}	ΔG_W	n_W
CO_2 (g)	-18.44	0.31	-0.69		
HCO_2H (aq)	-39.23	0.90	-0.70	-0.06	1
HCO_2^- (aq)	-79.38	0.54	-0.67	-0.19	4
$CH_2(OH)_2$ (aq)	-33.24	1.53	-0.73		0

Table S8. Free energy contributions (eV) for intermediates in the catalytic cycle.

	E_0	E_{ZPVE}	G_{trv}	ΔG_W	n_W
S_0	-102.27	4.41	-0.93	-0.10	1
S_1	-115.66	4.04	-0.89	-0.07	2
S_{15}	-162.23	4.90	-1.03	-0.63	4
S_{14}	-109.41	5.32	-1.03		0
S_{13}	-136.10	4.96	-1.02	-0.07	2
S_{12}	-135.92	5.32	-1.03	-0.06	2
S_{11}	-149.39	4.94	-1.02	-0.14	3
S_{18}	-196.12	5.80	-1.13	-0.66	5
S_{19}	-169.69	6.23	-1.12	-0.12	3
S_{20}	-169.77	5.85	-1.11	-0.15	3
S_{21}	-170.10	5.07	-1.05	-0.10	4
S_{22}	-130.14	5.40	-1.08	-0.09	1
S_{24}	-157.56	5.08	-1.06	-0.47	3
S_{25}	-143.90	5.45	-1.07	-0.07	2
S_{25}^*	-160.04	5.34	-1.07	-0.12	3
S_{26}^*	-147.12	5.73	-1.08	-0.16	2
S_{26}	-164.94	5.69	-1.07	-0.16	3
S_{27}	-151.48	6.07	-1.08	-0.07	2
S_{28}	-164.90	5.71	-1.06	-0.07	3
S_{29}	-138.16	6.07	-1.08	-0.02	1
S_{30}	-164.50	5.66	-1.08	-0.59	3

Table S9. Free energy contributions (eV) for transition states.

	E_0	E_{ZPVE}	G_{trv}	ΔG_W	n_W
$S_1 \rightarrow S_{15}$	-135.29	4.85	-1.08	-0.26	2
$S_{14} \rightarrow S_{13}$	-161.67	5.75	-1.02	-0.05	2
$S_{13} \rightarrow S_{12}$	-161.68	5.75	-1.03	-0.06	2
$S_{12} \rightarrow S_{11}$	-148.47	5.78	-1.03	-0.06	1
$S_{11} \rightarrow S_{18}$	-182.52	5.73	-1.25	-0.19	4
$S_{19} \rightarrow S_{20}$	-182.15	6.67	-1.12	-0.09	2

$S_{20}\rightarrow S_{21}$	-182.54	5.74	-1.22	-0.79	4
$S_{21}\rightarrow S_{22}$	-155.95	5.89	-1.06	-0.03	1
$S_{22}\rightarrow S_{24}$	-156.04	5.88	-1.07	-0.01	1
$S_{25}\rightarrow S_{25}^{*}$	-143.80	5.40	-1.07	-0.09	2
$S_{25}^{*}\rightarrow S_{26}^{*}$	-159.51	6.19	-1.07	-0.05	1
$S_{26}^{*}\rightarrow S_{26}$	-146.95	5.71	-1.07	-0.25	2
$S_{26}\rightarrow S_{27}$	-177.29	6.55	-1.08	-0.11	2
$S_{27}\rightarrow S_{28}$	-177.11	6.53	-1.07	-0.15	2
$S_{28}\rightarrow S_{29}$	-163.98	6.53	-1.07	-0.10	1
$S_{30}\rightarrow S_{1}$	-164.18	5.60	-1.16	-0.29	3

8. The pH dependence of the total barriers of all other steps in the catalytic cycle

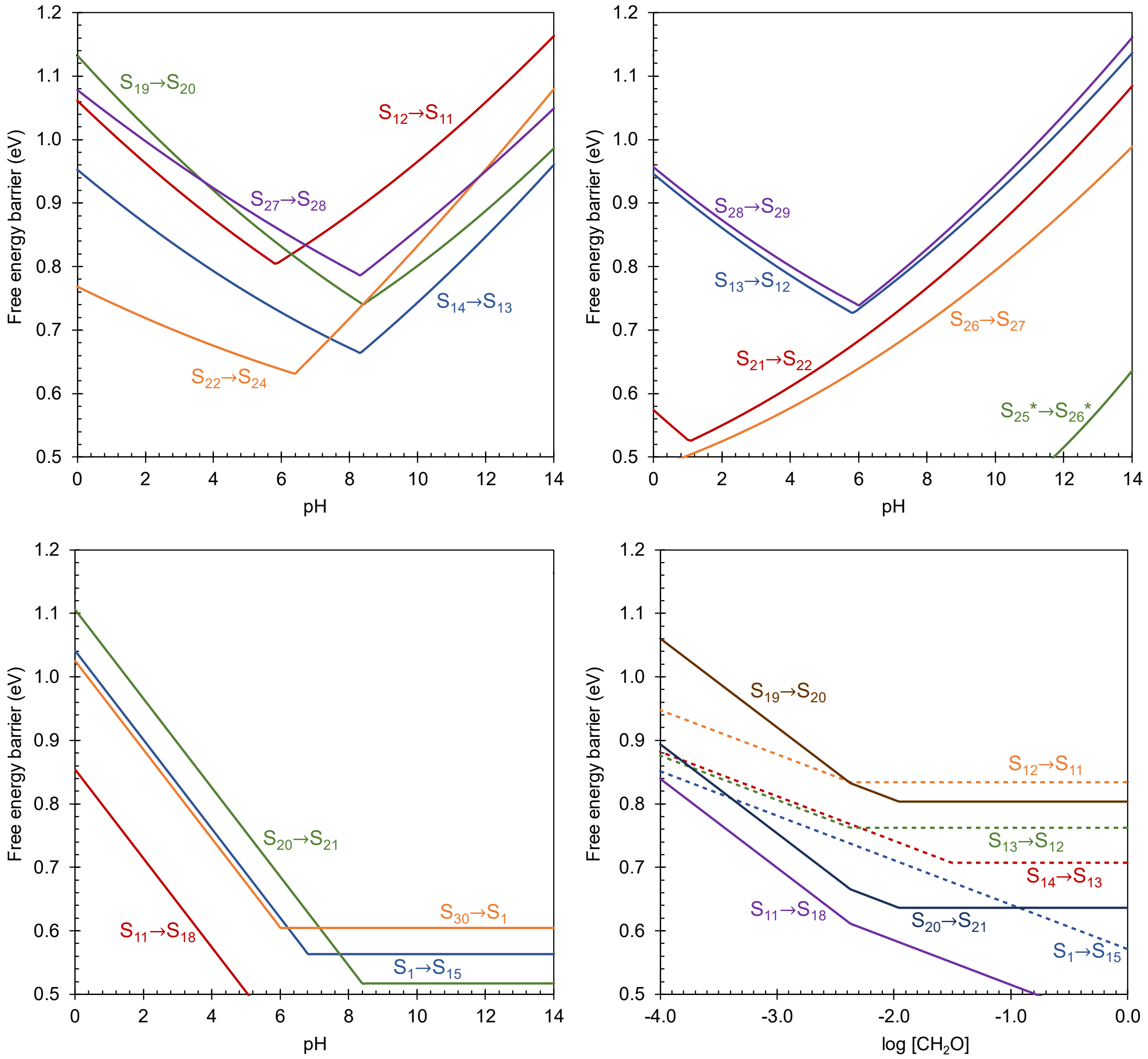

Figure S3. Total barriers for different steps in the chain growth cycle with respect to pH (at log[CH_2O]=0) and with respect to log[CH_2O] (at pH=6.7).

9. The kinetic dependence of the chain growth cycle on formaldehyde concentration at pH of 7.8

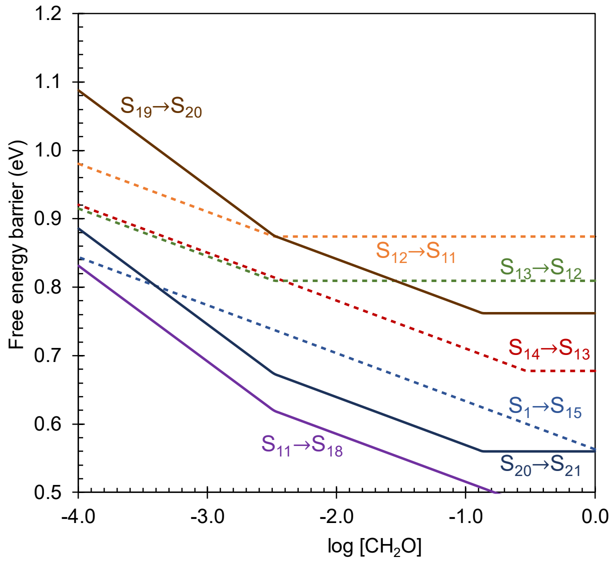

Figure S4. Total barriers for different steps in the chain growth cycle with respect to log[CH_2O] at pH=7.8.

10. The free energy diagrams for both the CO_2 reduction cycle and the chain growth cycle under optimal coupling conditions

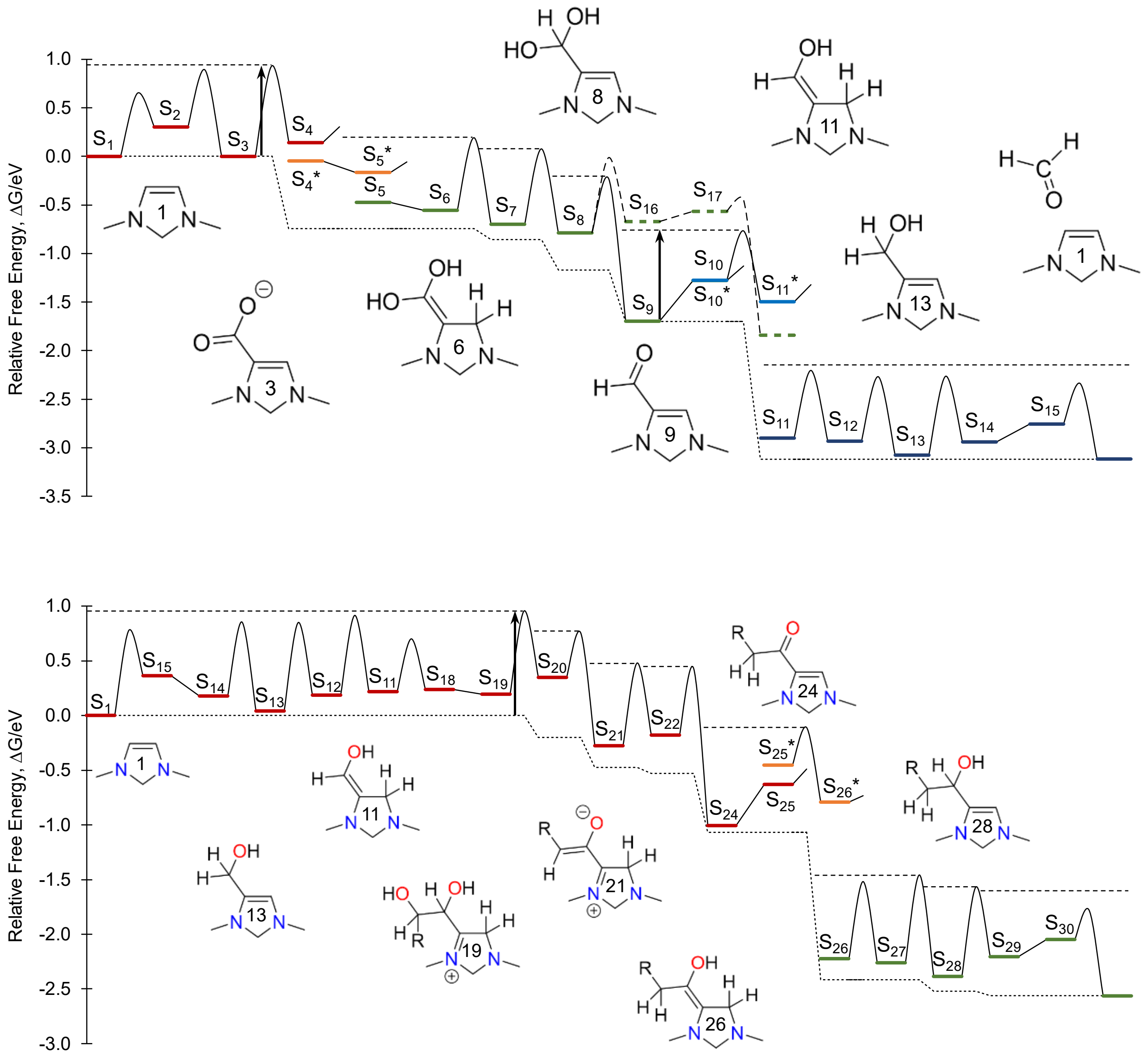

Figure S5. Free energy diagram for (top) CO_2 reduction to formaldehyde and (bottom) the chain growth cycle for formaldehyde to acetaldehyde at pH=7.8 and $[CH_2O]$ = 0.87 mmol

11. Structures and hydrogen bonding free energies of intermediates

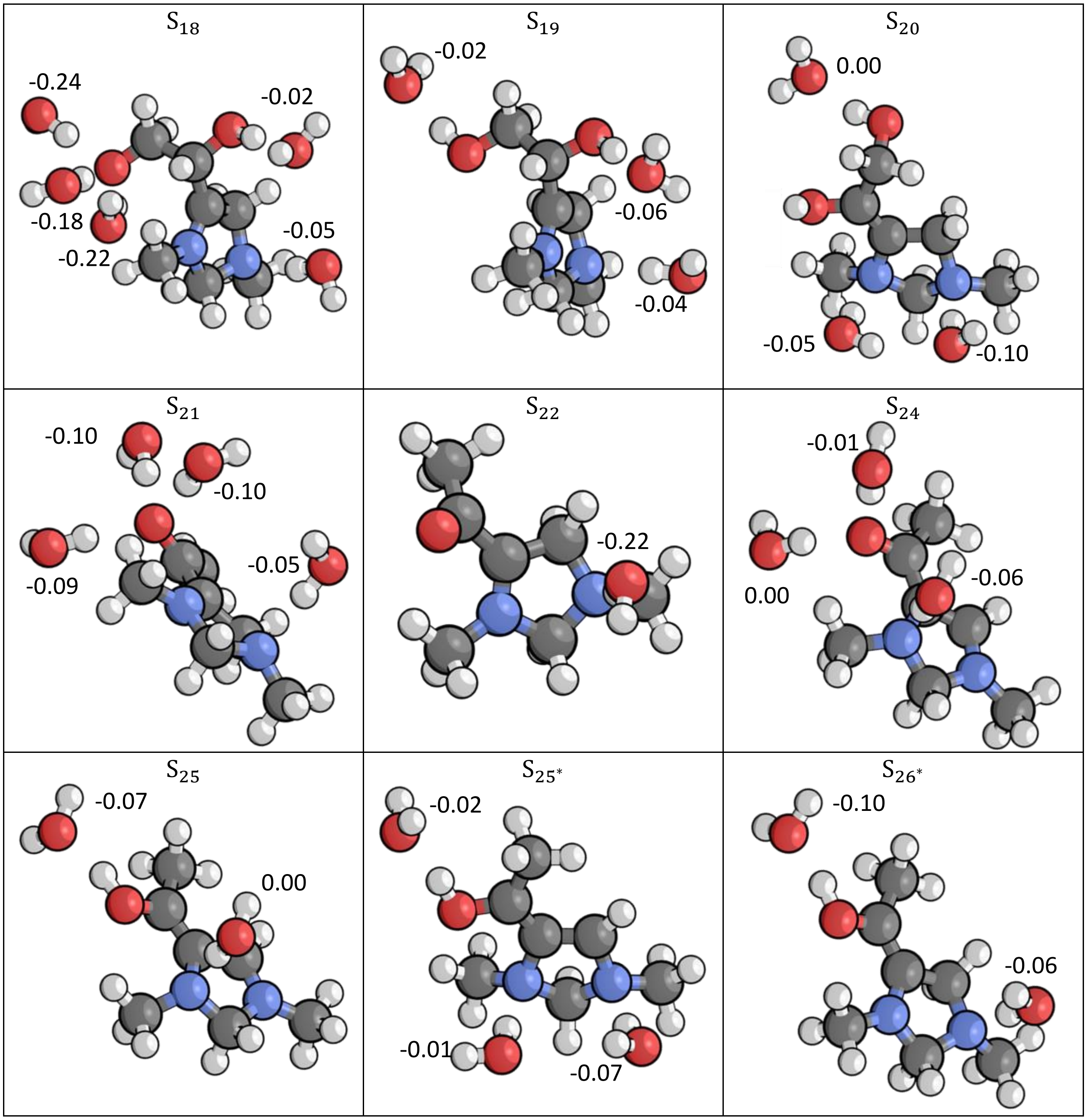

Figure S6. Structures and hydrogen bonding energies of intermediates in the catalytic cycle. The numerical values in each structure indicate the free energy (eV) associated with hydrogen bonding an explicit molecule of water at the associated position in the structure.

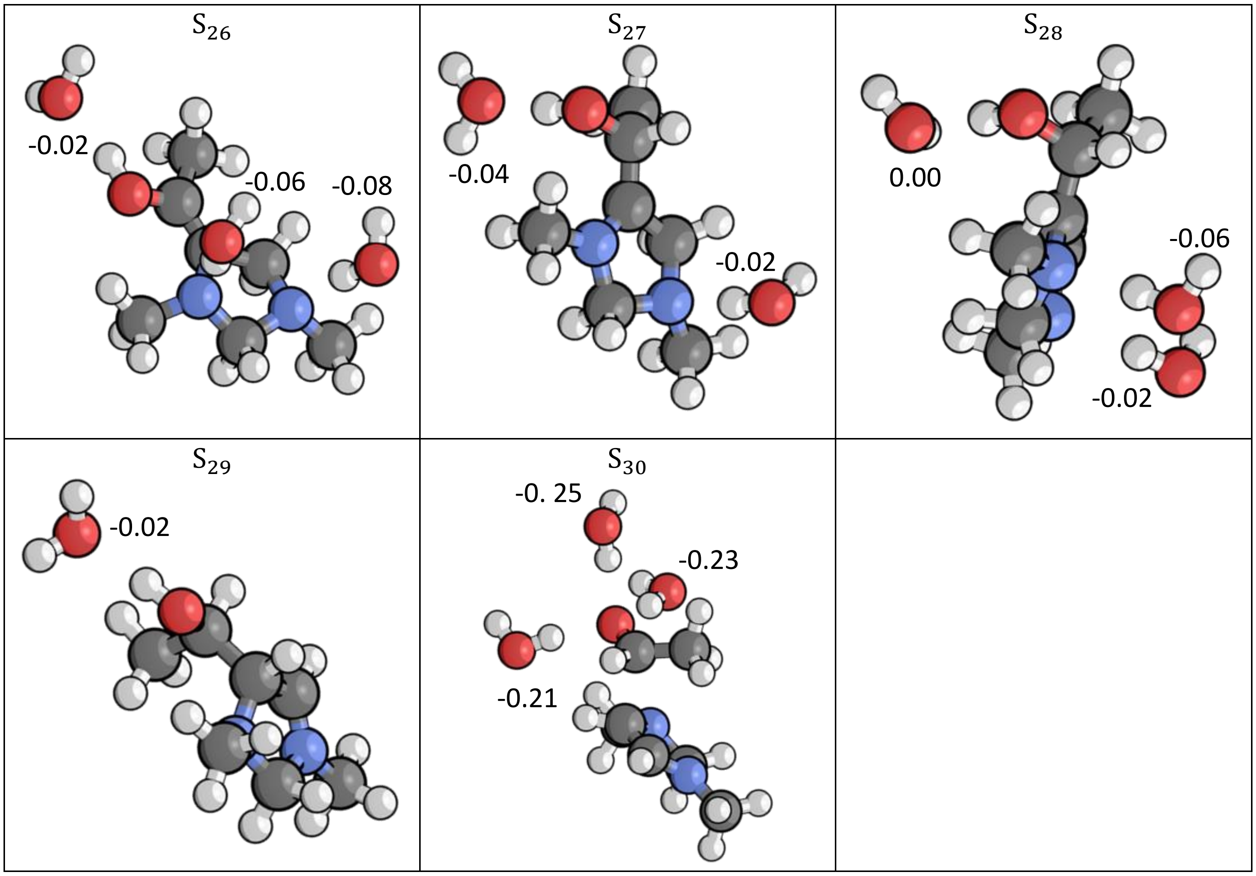

Figure S6 (continued). Structures and hydrogen bonding energies of intermediates in the catalytic cycle. The numerical values in each structure indicate the free energy (eV) associated with hydrogen bonding an explicit molecule of water at the associated position in the structure.

12. Structures and hydrogen bonding free energies of transition states

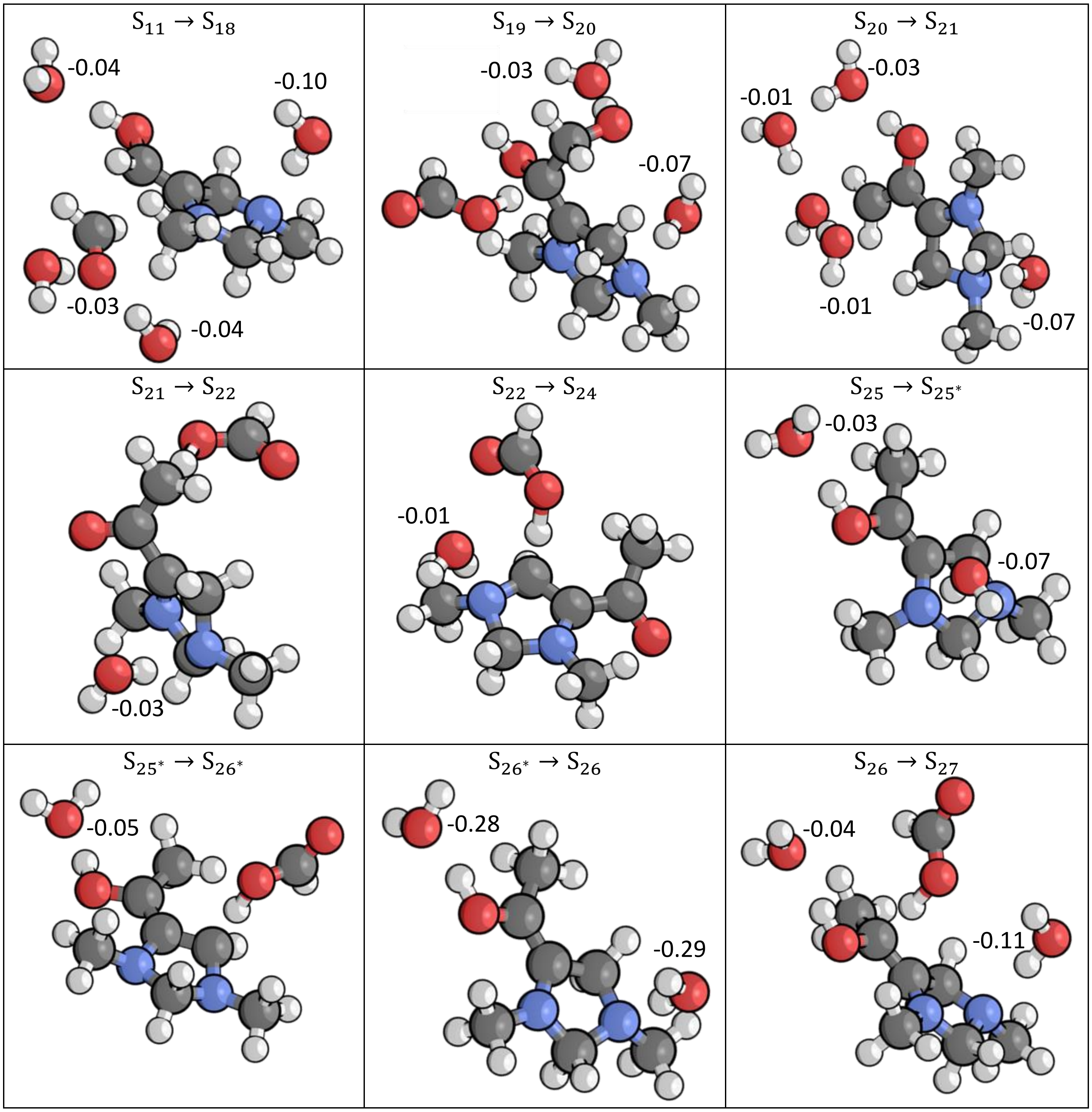

Figure S7. Structures and hydrogen bonding energies of transition states in the catalytic cycle. The numerical values in each structure indicate the free energy (eV) associated with hydrogen bonding an explicit molecule of water at the associated position in the structure.

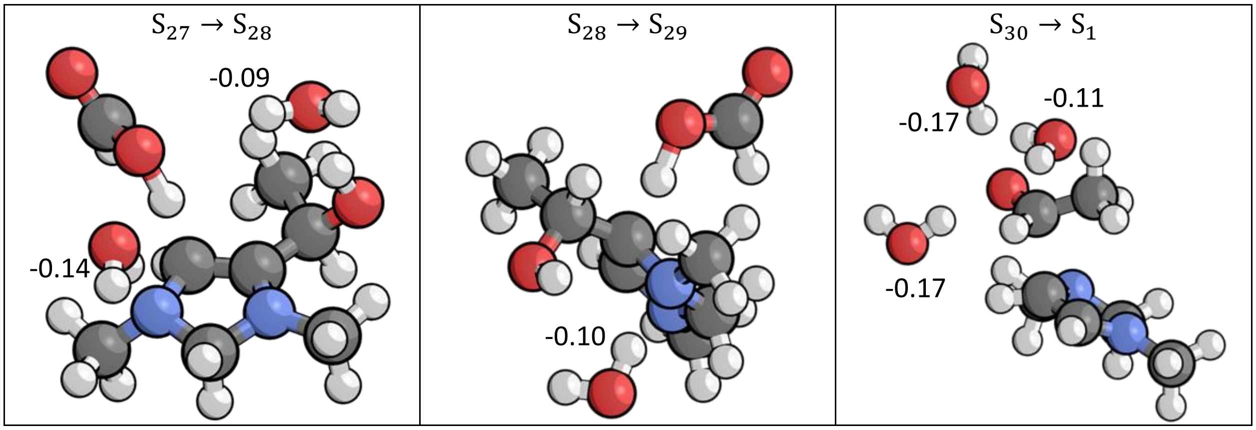

Figure S7 (continued). Structures and hydrogen bonding energies of transition states in the catalytic cycle. The numerical values in each structure indicate the free energy (eV) associated with hydrogen bonding an explicit molecule of water at the associated position in the structure.

References

(1) Khezeli, F.; Plaisance, C. Computational Design of an Electro-Organocatalyst for Conversion of CO2 into Formaldehyde. *J. Phys. Chem. A* **2024**, *128* (9), 1576–1592. https://doi.org/10.1021/acs.jpca.3c07806.

(2) Kresse, G.; Furthmüller, J. Efficient Iterative Schemes for Ab Initio Total-Energy Calculations Using a Plane-Wave Basis Set. *Phys. Rev. B* **1996**, *54* (16), 11169–11186. https://doi.org/10.1103/PhysRevB.54.11169.

(3) Mathew, K.; Kolluru, V. S. C.; Mula, S.; Steinmann, S. N.; Hennig, R. G. Implicit Self-Consistent Electrolyte Model in Plane-Wave Density-Functional Theory. *The Journal of Chemical Physics*, 2019, *151*, 234101. https://aip.scitation.org/doi/abs/10.1063/1.5132354.

(4) Mathew, K.; Sundararaman, R.; Letchworth-Weaver, K.; Arias, T. A.; Hennig, R. G. Implicit Solvation Model for Density-Functional Study of Nanocrystal Surfaces and Reaction Pathways. *The Journal of Chemical Physics*, 2014, *140*, 084106. https://doi.org/10.1063/1.4865107.

(5) Wellendorff, J.; Lundgaard, K. T.; Møgelhøj, A.; Petzold, V.; Landis, D. D.; Nørskov, J. K.; Bligaard, T.; Jacobsen, K. W. Density Functionals for Surface Science: Exchange-Correlation Model Development with Bayesian Error Estimation. *Phys. Rev. B* **2012**, *85* (23), 235149. https://doi.org/10.1103/PhysRevB.85.235149.

(6) Blöchl, P. E. Projector Augmented-Wave Method. *Phys. Rev. B* **1994**, *50* (24), 17953–17979. https://doi.org/10.1103/PhysRevB.50.17953.

(7) Kresse, G.; Joubert, D. From Ultrasoft Pseudopotentials to the Projector Augmented-Wave Method. *Phys. Rev. B* **1999**, *59* (3), 1758–1775. https://doi.org/10.1103/PhysRevB.59.1758.

(8) Henkelman, G.; Jónsson, H. A Dimer Method for Finding Saddle Points on High Dimensional Potential Surfaces Using Only First Derivatives. *The Journal of Chemical Physics*, 1999, *111*, 7010–7022.

(9) Henkelman, G.; Uberuaga, B. P.; Jónsson, H. A Climbing Image Nudged Elastic Band Method for Finding Saddle Points and Minimum Energy Paths. *The Journal of Chemical Physics*, 2000, *113*, 9901–9904.

(10) Henkelman, G.; Jónsson, H. Improved Tangent Estimate in the Nudged Elastic Band Method for Finding Minimum Energy Paths and Saddle Points. *The Journal of Chemical Physics*, 2000, *113*, 9978–9985.

(11) Celani, P.; Robb, M. A.; Garavelli, M.; Bernardi, F.; Olivucci, M. Geometry Optimisation on a Hypersphere. Application to Finding Reaction Paths from a Conical Intersection. *Chemical Physics Letters* **1995**, *243* (1), 1–8. https://doi.org/10.1016/0009-2614(95)00821-K.

(12) Valiev, M.; Bylaska, E. J.; Govind, N.; Kowalski, K.; Straatsma, T. P.; Van Dam, H. J. J.; Wang, D.; Nieplocha, J.; Apra, E.; Windus, T. L.; de Jong, W. A. NWChem: A Comprehensive and Scalable Open-Source Solution for Large Scale Molecular Simulations. *Computer Physics Communications* **2010**, *181* (9), 1477–1489. https://doi.org/10.1016/j.cpc.2010.04.018.

(13) Adamo, C.; Cossi, M.; Barone, V. An Accurate Density Functional Method for the Study of Magnetic Properties: The PBE0 Model. *Journal of Molecular Structure: THEOCHEM* **1999**, *493* (1), 145–157. https://doi.org/10.1016/S0166-1280(99)00235-3.

(14) Becke, A. D. Density-functional Thermochemistry. III. The Role of Exact Exchange. *The Journal of Chemical Physics* **1993**, *98* (7), 5648–5652. https://doi.org/10.1063/1.464913.

(15) Klamt, A.; Schüürmann, G. COSMO: A New Approach to Dielectric Screening in Solvents with Explicit Expressions for the Screening Energy and Its Gradient. *J. Chem. Soc., Perkin Trans. 2* **1993**, No. 5, 799–805. https://doi.org/10.1039/P29930000799.

(16) York, D. M.; Karplus, M. A Smooth Solvation Potential Based on the Conductor-Like Screening Model. *J. Phys. Chem. A* **1999**, *103* (50), 11060–11079. https://doi.org/10.1021/jp992097l.

(17) Perdew, J. P.; Burke, K.; Wang, Y. Generalized Gradient Approximation for the Exchange-Correlation Hole of a Many-Electron System. *Phys. Rev. B* **1996**, *54* (23), 16533–16539. https://doi.org/10.1103/PhysRevB.54.16533.
(18) Seyedraoufi, S.; Berland, K. Improved Proton-Transfer Barriers with van Der Waals Density Functionals: Role of Repulsive Non-Local Correlation. *The Journal of Chemical Physics* **2022**, *156* (24), 244106. https://doi.org/10.1063/5.0095128.
